# Biomarker Quantification, Spectroscopic, and Molecular Docking Studies of the Active Compounds Isolated from the Edible Plant *Sisymbrium irio* L.

**DOI:** 10.3390/ph16040498

**Published:** 2023-03-27

**Authors:** Shaza M. Al-Massarani, Latifah S. Aldurayhim, Ibtisam A. Alotaibi, Mostafa W. M. Abdelmageed, Md Tabish Rehman, Omer A. Basudan, Maged S. Abdel-Kader, Mohamed F. Alajmi, Fatma M. Abdel Bar, Perwez Alam, Maram M. Al Tamimi, Ali A. El Gamal

**Affiliations:** 1 Department of Pharmacognosy, College of Pharmacy, King Saud University, P.O. Box 2457, Riyadh 11451, Saudi Arabia; 2 Pharmacognosy Department, College of Pharmacy, Sattam Bin Abdulaziz University, Al-Kharj 11942, Saudi Arabia; 3 Department of Pharmacognosy, College of Pharmacy, Alexandria University, Alexandria 21215, Egypt; 4 Pharmacognosy Department, Faculty of Pharmacy, Mansoura University, El Mansoura 35516, Egypt

**Keywords:** *Sisymbrium irio*, unsaturated fatty acids, indole alkaloids, molecular docking, HPTLC standardization

## Abstract

Phytochemical investigation of the ethanolic extract of the aerial parts of *Sisymbrium irio* L. led to the isolation of four unsaturated fatty acids (**1**–**4**), including a new one (**4**), and four indole alkaloids (**5**–**8**). The structures of the isolated compounds were characterized with the help of spectroscopic techniques such as 1D, 2D NMR, and mass spectroscopy, and by correlation with the known compounds. In terms of their notable structural diversity, a molecular docking approach with the AutoDock 4.2 program was used to analyze the interactions of the identified fatty acids with PPAR-γ and the indole alkaloids with 5-HT_1A_ and 5-HT_2A_, subtypes of serotonin receptors, respectively. Compared to the antidiabetic drug rivoglitazone, compound **3** acted as a potential PPAR-γ agonist with a binding energy of −7.4 kcal mol^−1^. Moreover, compound **8** displayed the strongest affinity, with binding energies of −6.9 kcal/mol to 5HT_1A_ and −8.1 kcal/mol to 5HT_2A_, using serotonin and the antipsychotic drug risperidone as positive controls, respectively. The results of docked conformations represent an interesting target for developing novel antidiabetic and antipsychotic drugs and warrant further evaluation of these ligands in vitro and in vivo. On the other hand, an HPTLC method was developed to quantify α-linolenic acid in the hexane fraction of the ethanol extract of *S. irio*. The regression equation/correlation coefficient (r^2^) for linolenic acid was Y = 6.49X + 2310.8/0.9971 in the linearity range of 100–1200 ng/band. The content of α-linolenic acid in *S. irio* aerial parts was found to be 28.67 μg/mg of dried extract.

## 1. Introduction

Plants of the mustard family (Brassicaceae), such as broccoli, mustard, cabbage, cauliflower, kale, and turnip, have dietary value, economic importance, and traditional medicinal uses [1]. *Sisymbrium* is a member of the family Brassicaceae with around 90 species endemic to temperate Asia, Europe, the Mediterranean, southern Africa, and Australia. Some of these species were used in folkloric medicine to treat bronchitis, stomach ailments, voice disorders, sore throats, and as poison antidotes [2,3]. Previous phytochemical studies on *Sisymbrium* have led to the isolation of glucosinolate glycosides, flavonoids, alkaloids, anthraquinones, and steroids [3,4,5].

*Sisymbrium irio* L. is an edible yellow-flowered plant, native to Asia, North Africa, and southern Europe, and has been transferred by migrants, either accidentally or intentionally, to profuse areas around the world. It is classified as an invasive and notorious weed in several countries [3]. Like most Brassicaceae plants, *S. irio* is well-known for its highly nutritional value and rich content of protein, minerals, fatty acids, and vitamins, both in the seeds and aerial parts [6]. Moreover, it is used in folkloric medicine to treat different ailments due to its antioxidant, antimicrobial, anti-inflammatory, and diuretic properties [7,8,9].

Rivoglitazone, together with rosiglitazone and pioglitazone are thiazolidinedione-derivatives. They act by binding to the nuclear receptor peroxisome proliferator-activated receptor gamma (PPAR-γ). Rivoglitazone, reported as the most potent PPAR-γ agonist, decreases plasma glucose and triglyceride levels in a dose-dependent manner in animals. The efficacy and safety of rivoglitazone for the treatment of type 2 diabetes mellitus patients have been proven by stage II and stage III clinical studies [10].

Serotonin, 5-HT (5-hydroxytryptamine), is a well-known neurotransmitter due to its vital role in many physiological functions, such as sleep, appetite, and pain perception in several pathological disorders, including migraine, depression, and anxiety [11]. Thus, 5-HT receptors are important therapeutic targets for the treatment of several CNS conditions. On the other hand, risperidone is an atypical antipsychotic medication. It acts as an antagonist for the serotonin 5-HT_2A_ receptor with high affinity, leading to serotonin and norepinephrine reuptake inhibition. It has also been used as an adjunct for severe depression and in the treatment of non-psychotic unipolar depression [12].

The long traditional history of ‘plant-based therapies’ has always been a motivation to investigate the potential pharmacological activities of the isolated secondary metabolites through molecular docking, a cost-effective and reliable computational methodology that has been a valuable tool to discover novel drug candidates [13,14].

In the current study, we describe the isolation and identification of eight compounds belonging to two classes (unsaturated fatty acids and indole alkaloids) and perform comparative molecular docking and binding free energy calculations to rank the identified compounds based on their binding affinities with the ligand-binding domains (LBD) of the therapeutic targets PPAR-γ and 5-HT_1A_ and 5-HT_2A_, respectively. We also developed a sensitive HPTLC method for the quantification of the biomarker compound α-linolenic acid in the ethanol extract of *S. irio*.

## 2. Results

### 2.1. Identification of Isolated Compounds

**Figure 1:**
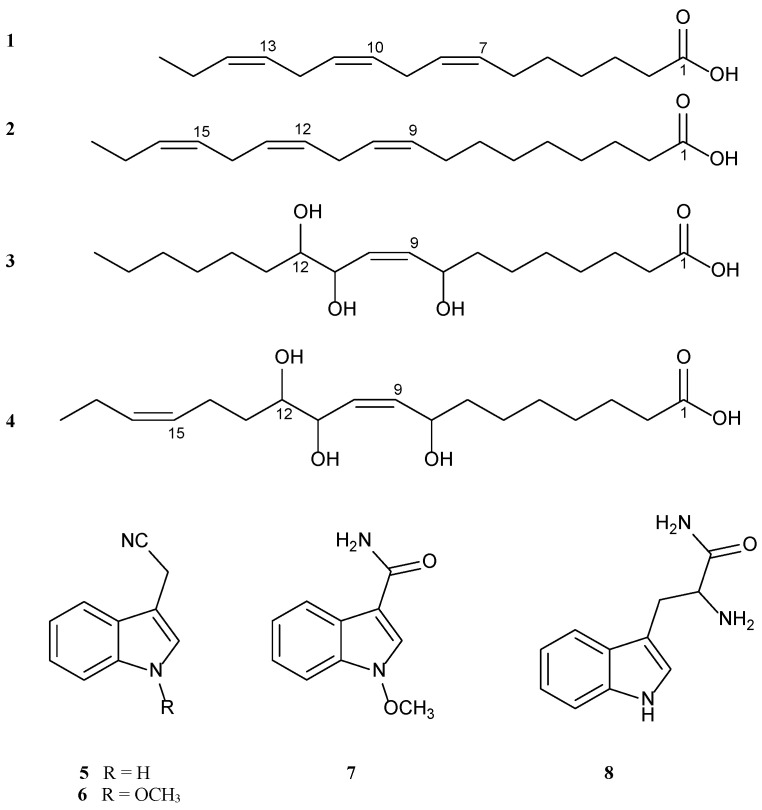
Chemical structures of the isolated compounds (**1**–**8**) from *Sisymbrium irio* L.

The structures of the isolated compounds (Figure 1) were elucidated by 1D, 2D NMR analyses, MS, and by comparing with the literature data (Tables S1 and S2) [15,16,17,18,19,20,21].

Three known fatty acids (**1**–**3**) were identified as (7*Z*,10*Z*,13*Z*)-hexadecatrienoic acid (roughanic acid) (RA) (**1**) [15], (9*Z*,12*Z*,15*Z*)-octadecatrienoic acid (α-linolenic acid) (**2**) [16,17], and 8,11,12-trihydroxy-9*Z*-octadecanoic acid (**3**) [18], and the indole alkaloids (**5**–**8**) were identified as 1*H*-indole-3-acetonitrile (**5**) [19], 1-methoxyindole 3-acetonitrile (**6**) [19], 1-methoxy-1*H*-indole-3-carboxamide (**7**) [20], and α-amino-3-indole propanamide (L-tryptophanamide) (**8**) [21].

Compound **4** was isolated as a white amorphous solid. The HRESIMS showed quasi-molecular ion peaks at m/z 329.2325 [M+H]^+^ and 351.2140 [M+Na]^+^, consistent with a molecular weight of 328 amu, and a molecular formula of C_18_H_32_O_5_. IR absorption at 3315, 1697, and 1455 cm^−1^ indicated the presence of hydroxyl group, carboxylic group, and olefinic double bonds, respectively.

The ^13^C NMR spectrum and DEPT experiment in CD_3_OD revealed **4** to be C_18_-aliphatic acid, comprising one carboxylic group: C-1 (δ_C_ 177.9); four *Sp^2^* methines forming two double bonds: C-9 (δ_C_ 136.5), C-10 (δ_C_ 131.0), C-15 (δ_C_ 126.3), and C-16 (δ_C_ 134.4); three oxygen-bearing *Sp3* methines: C-8 (δ_C_ 73.0), C-11 (δ_C_ 75.8), and C-12 (δ_C_ 75.7); nine methylenes; and one methyl group: C-18 (δ_C_ 14.6) (Table 1). Furthermore, the ^1^H NMR spectrum showed a terminal methyl group: H_3_-18 (δ_H_ 0.85, t, *J* = 7.4 Hz); one methylene triplet: H_2_-2 (δ_H_ 2.16, t, *J* = 7.3 Hz); three oxygenated methines: H-8 (δ_H_ 3.98, m), H-11 (δ_H_ 3.85, t, *J* = 6.0 Hz), and H-12 (δ_H_ 3.38, m); and four olefinic protons of two *cis*-ene functions H-9/10 at (δ_H_ 5.60, m) and H-15/16 at (δ_H_ 5.34, m) (Table 1).

**Figure 2:**
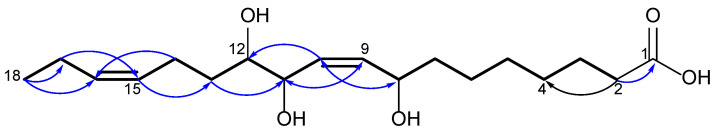
^1^H-^1^H COSY (―) and key ^1^H-^13^C HMBC (H→C) correlations of compound **4**.

The combined analysis of the COSY and HSQC spectra of **4** allowed establishing a continuous chain of carbon atoms from C-2 to C-18, which was further corroborated by HMBC correlations as shown in Figure 2. Moreover, the locations of hydroxyl groups and double bonds were also identified using COSY and HMBC experiments and confirmed at C-8, C-11, and C-12 for the three hydroxyl groups; and at C-9/10 and C-15/16 for the two olefinic double bonds. The *Z*-configuration of the double bonds was judged from their small coupling constant (*J* < 10 Hz) compared with the reported *E*-configuration [22,23].

Furthermore, the HMBC spectrum revealed key correlations between H_2_-2 (δ_H_ 2.16) and C-1 (δ_C_ 177.9) and C-4 (δ_C_ 30.1); H-8 (δ_H_ 3.98) and C-10 (δ_C_ 131.0); H-9 (δ_H_ 5.60) and C-11 (δ_C_ 75.8); H-10 (δ_H_ 5.60) and C-12 (δ_C_ 75.7); H-15 (δ_H_ 5.34) and C-13 (δ_C_ 31.5); H-14 (δ_H_ 1.22) and C-16 (δ_C_ 134.4); H-17 (δ_H_ 1.95) and C-15 (δ_C_ 126.3); and H_3_-18 (δ_H_ 0.85) and C-16 (δ_C_ 134.4) and C-17 (δ_C_ 21.6) (Figure 2). Accordingly, the structure of **4** was identified as 8,11,12-trihydroxy-9*Z*,15*Z*-octadecadienoic acid.

### 2.2. Analysis of Molecular Docking

#### 2.2.1. Molecular Docking of Fatty Acids (**1**–**4**) with PPAR-γ

**Figure 3:**
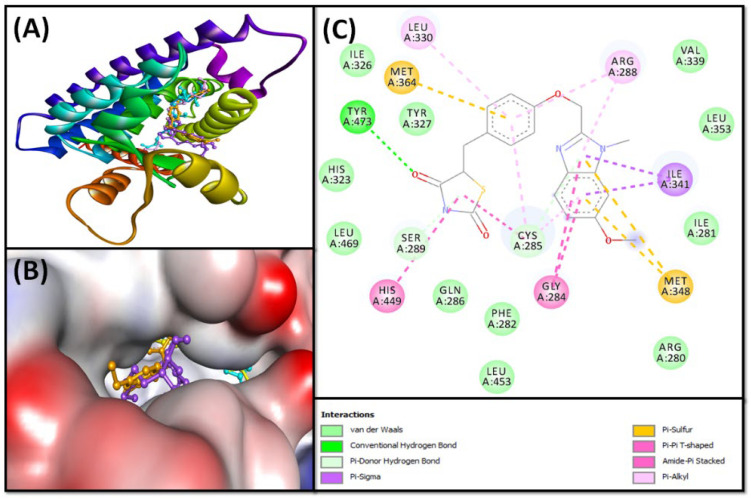
Molecular docking of isolated SI fatty acids and rivoglitazone (control) with PPAR-γ. (**A**) Two-dimensional representation of ligands binding to the protein; (**B**) three-dimensional representation of ligands binding at the cavity of the protein; and (**C**) molecular interaction and the amino acid residues involved in stabilizing rivoglitazone (control) and PPAR-γ complex formation.

**Figure 4:**
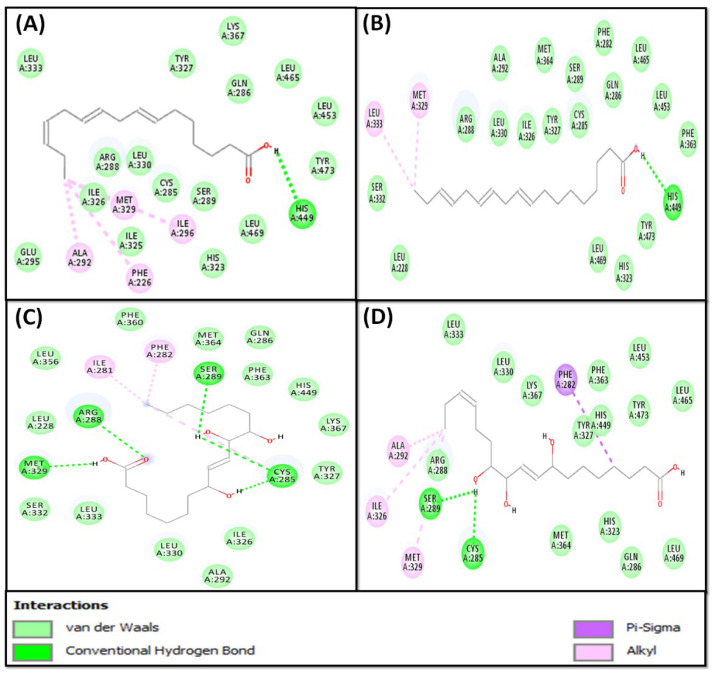
Molecular interaction between isolated SI fatty acids and PPAR-γ. (**A**) Compound **1**, (**B**) compound **2**, (**C**) compound **3**, and (**D**) compound **4**.

The isolated fatty acids (**1**–**4**) from *S. irio* were subjected to molecular docking experiments with peroxisome proliferator-activated receptor gamma (PPAR-γ or PPARG), also known as the glitazone reverse insulin resistance receptor, based on the reported antidiabetic activity for similar compounds [24,25,26,27]. The relative binding of fatty acids to the PPAR-γ substrate-binding site is described in Table 2 and Figure 3 and Figure 4. The details of the protein–ligand interaction are presented in Supplementary Table S3.

It is obvious that all isolated fatty acids have a docking energy in the range of −6.0 to −7.4 kcal/mol (Table 2), and the lowest binding energy was revealed by compound **3**. To strengthen our finding, the binding potentials of (**1**–**4**) were compared with rivoglitazone as a positive control. The details of interactions between PPAR-γ and fatty acids, along with rivoglitazone, are discussed in the subsequent sections.

The analysis revealed that the PPAR-γ–rivoglitazone complex was stabilized by hydrophobic bonding (Figure 3A,B), including two Pi-Sigma hydrophobic bonds with ILE^341^ and three amide-Pi-stacked interactions with GLY^284^ (two interactions) and CYS^285^ (one interaction). Further, there were five Pi–Alkyl hydrophobic interactions with CYS^285^ (two interactions), ARG^288^ (two interactions), and LEU^330^ (one interaction); and one Pi–Pi T-shaped hydrophobic interaction with HIS^449^ (Figure 3C). In addition, the protein–ligand complex was stabilized by three Pi–S bonds with MET^348^ (two interactions) and MET^364^ (one interaction); two Pi-donor hydrogen bonds with CYS^285^ and SER^289^; and one conventional hydrogen bond with TYR^473^. The van der Waals interactions were also present with amino acid residues like ARG^280^, ILE^281^, PHE^282^, GLN^286^, HIS^323^, ILE^326^, TYR^327^, VAL^339^, LEU^353^, LEU^453^, and LEU^469^. The PPAR-γ–rivoglitazone complex was stabilized by an estimated free energy of −8.2 kcal mol^−1^, which corresponds to a dissociation constant of 1.03 × 10^6^ M^−1^ (Table 2).

Furthermore, compound **1** was found to bind at the central binding cavity of PPAR-γ and the compound **1**–PPAR-γ complex was mainly stabilized by hydrophobic interactions (Figure 4A). Compound **1** formed one hydrogen bond with HIS^449^ and four hydrophobic (alkyl) interactions with PHE^226^, ALA^292^, ILE^296^, and MET^329^ (Figure 4A). In addition, compound **1** formed van der Waals interactions with CYS^285^, GLN^286^, ARG^288^, SER^289^, GLU^295^, HIS^323^, ILE^325^, ILE^326^, TYR^327^, LEU^330^, LEU^333^, LYS^367^, LEU^453^, LEU^465^, LEU^469^, and TYR^473^. The binding energy and dissociation constant for the compound 1–PPAR-γ interaction were −6.7 kcal mol^−1^ and 8.21 × 10^4^ M^−1^, respectively (Table 2).

The analysis of molecular docking also suggests that compound **2** occupied the active site of PPAR-γ (Figure 4B). The compound **2**–PPAR-γ complex was stabilized by one hydrogen bond with HIS^449^ and two hydrophobic (alkyl) interactions with MET^329^ and LEU^333^ (Figure 4B). In addition, compound **2** formed van der Waals interactions with LEU^228^, PHE^282^, CYS^285^, ALA^292^, GLN^286^, ARG^288^, SER^289^, HIS^323^, TYR^327^, LEU^330^, SER^332^, ILE^326^, PHE^363^, MET^364^, LEU^453^, LEU^465^, LEU^469^, and TYR^473^. Gibb’s free energy of the complex formation was −6.0 kcal mol^−1^, which corresponds to a dissociation constant of 2.52 × 10^4^ M^−1^ (Table 2).

The compound **3**–PPAR-γ complex was mainly stabilized by hydrogen bonds and hydrophobic interactions. Compound **3** formed five hydrogen bonds with CYS^285^ (two bonds), ARG^288^, SER^289^, and MET^329^. It also interacted with PPAR-γ through three hydrophobic (alkyl) interactions with ILE^281^, PHE^282^, and CYS^285^ (Figure 4C). In addition, compound **3** formed van der Waals interactions with LEU^228^, GLN^286^, ALA^292^, ILE^326^, TYR^327^, LEU^330^, SER^332^, LEU^333^, LEU^356^, PHE^360^, PHE^363^, MET^364^, LYS^367^, and HIS^449^. The binding energy and dissociation constant for the compound 3–PPAR-γ interaction were −7.4 kcal mol^−1^ and 2.68 × 10^5^ M^−1^, respectively (Table 2).

Finally, the analysis of the compound **4**–PPAR-γ interaction revealed that the complex was stabilized by hydrogen bonds as well as hydrophobic (alkyl) interactions. Compound **4** formed two hydrogen bonds with CYS^285^ and SER^289^ and one Pi–Sigma bond with PHE^282^. Moreover, compound **4** interacted hydrophobically (alkyl) with ALA^292^, ILE^326^, and MET^329^ (Figure 4D). In addition, compound **4** formed van der Waals interactions with GLN^286^, ARG^288^, HIS^323^, TYR^327^, LEU^330^, LEU^333^, PHE^363^, MET^364^, LYS^367^, HIS^449^, LEU^453^, LEU^465^, LEU^469^, and TYR^473^. Gibb’s free energy of the complex formation was −6.1 kcal mol^−1^, which corresponds to a dissociation constant of 2.98 × 10^4^ M^−1^ (Table 2).

#### 2.2.2. Molecular Docking of Compounds **5**–**8** with 5-HT_1A_ and 5-HT_2A_ Serotonin Receptors

**Figure 5:**
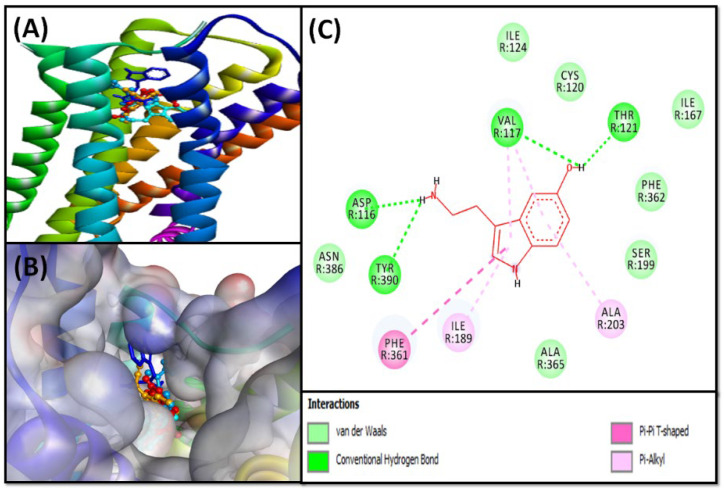
Molecular docking of isolated SI indole compounds and serotonin with 5-HT_1A_. (**A**) Two-dimensional representation of ligands binding to the protein; (**B**) three-dimensional representation of ligands binding at the cavity of the protein; and (**C**) molecular interaction and the amino acid residues involved in stabilizing serotonin (control) and 5-HT_1A_ complex formation.

**Figure 6:**
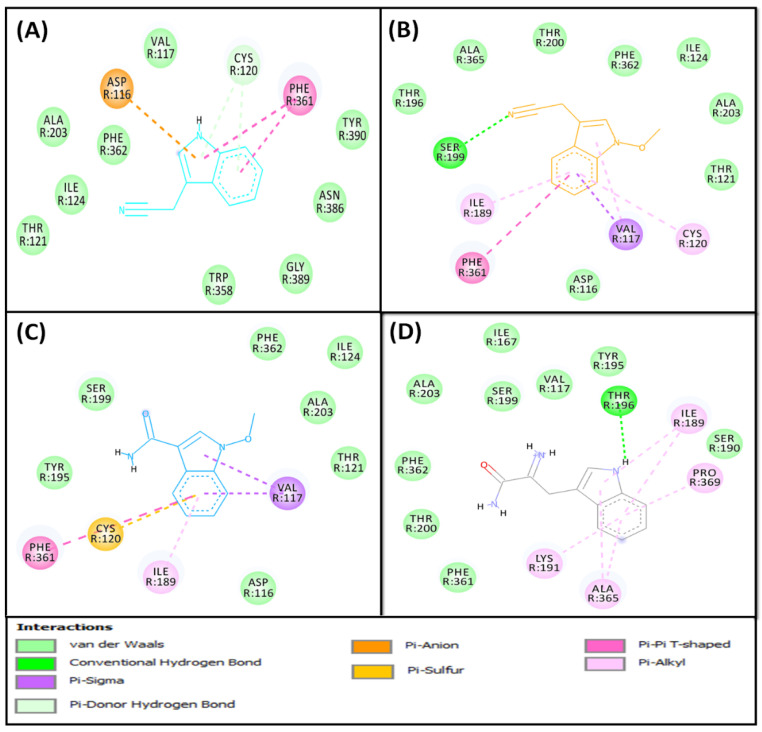
Molecular interaction between SI indole compounds and 5-HT_1A_. (**A**) Compound **5**, (**B**) compound **6**, (**C**) compound **7**, and (**D**) compound **8**.

**Figure 7:**
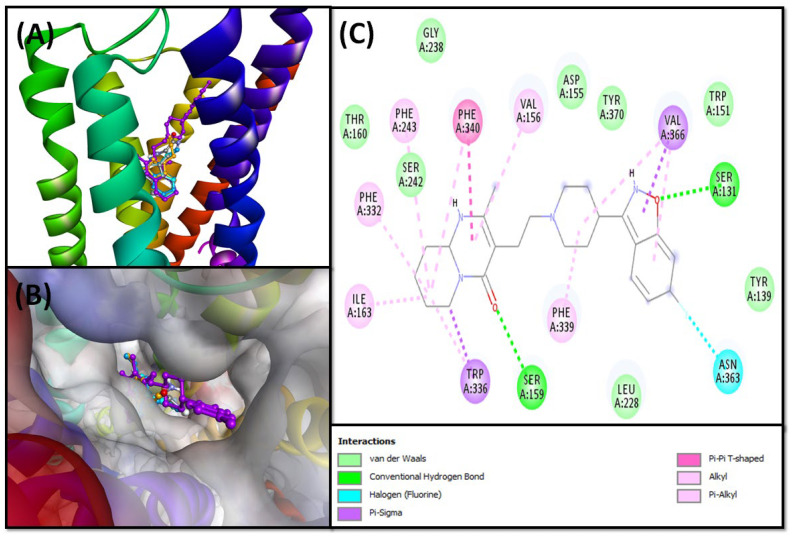
Molecular docking of isolated SI indole compounds and resperidone with 5-HT_2A_. (**A**) Two-dimensional representation of ligands binding to the protein; (**B**) three-dimensional representation of ligands binding at the cavity of the protein; and (**C**) molecular interaction and the amino acid residues involved in stabilizing resperidone (control) and 5-HT_2A_ complex formation.

**Figure 8:**
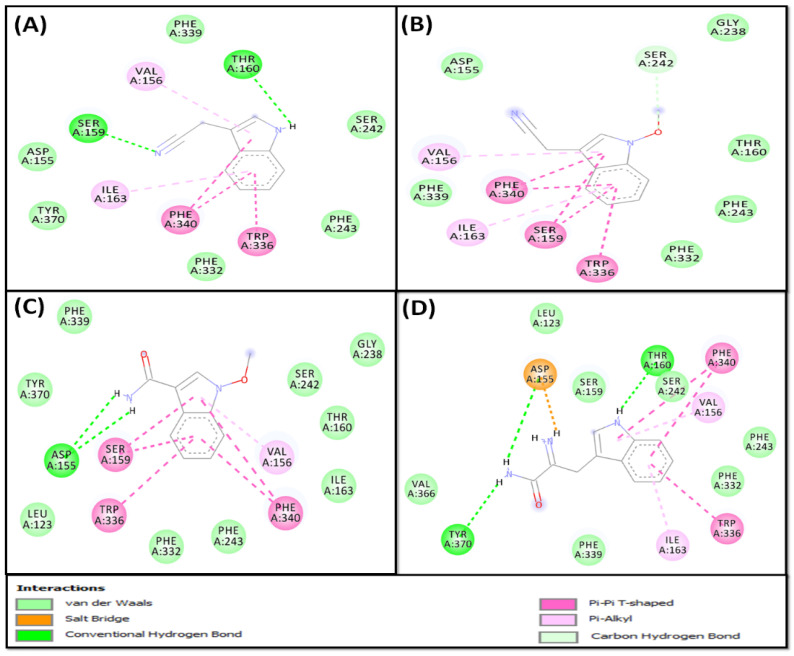
Molecular interaction between *S. irio* compounds and 5-HT_2A_. (**A**) Compound **5**, (**B**) compound **6**, (**C**) compound **7**, and (**D**) compound **8**.

Indole compounds (**5**–**8**) were screened for antidepressant activity using 5-HT_1A_ and 5-HT_2A_ serotonin receptors as potential targets. The relative binding of indole compounds is described in Table 3 and Table 4 and Figure 5, Figure 6, Figure 7 and Figure 8, and the detailed protein–ligand interaction is presented in Supplementary Tables S4 and S5.

The results exhibited that all indole compounds (**5**–**8**) were able to bind to the substrate binding site of 5-HT_1A_ (Figure 5A,B) and 5-HT_2A_ (Figure 7A,B) receptors, with more affinity towards the 5-HT_2A_ receptor, and their binding energies varied in the range between −6.4 and −6.9 kcal mol^−1^ and between −7.3 and −8.1 kcal mol^−1^, respectively. The binding potentials of indole compounds towards 5-HT_1A_ and 5-HT_2A_ receptors were compared with serotonin and risperidone, respectively, as positive controls (Table 3 and Table 4). The details of interactions between indole compounds and 5-HT_1A_ and 5-HT_2A_, along with positive controls, are discussed in the subsequent sections.

##### 5-HT_1A_ Binding Interaction

The molecular docking of serotonin (positive control) showed that it occupied the active site of 5-HT_1A_ and interacted primarily through hydrogen bonds and hydrophobic interactions (Figure 5A,B). It formed four hydrogen bonds with ASP^116^, VAL^117^, THR^121^, and TYR^390^; one Pi–Pi T-shaped hydrophobic interaction with PHE^361^; and four Pi–Alkyl hydrophobic interactions with VAL^117^ (two interactions), ILE^189^, and ALN^203^ (Figure 5C). In addition, the protein–ligand complex was stabilized by van der Waals interactions with CYS^120^, ILE^124^, ILE^167^, SER^199^, PHE^362^, ALA^365^, and ASN^386^. The 5-HT_1A_–serotonin complex was stabilized by −6.1 kcal mol^−1^ free energy, which corresponds to a dissociation constant of 6.1 × 10^4^ M^−1^ (Table 3).

The analysis of the 5-HT_1A_–**5** interaction revealed that the complex was stabilized by an electrostatic (Pi–Anion) interaction with ASP^116^, two hydrogen bonds with CYS^120^, and two Pi–Pi T-shaped hydrophobic interactions with PHE^361^ (Figure 6A). In addition, it formed van der Waals interactions with VAL^117^, THR^121^, ILE^124^, ALA^203^, TRP^358^, PHE^362^, ASN^386^, GLY^389^, and TYR^390^. Gibb’s free energy of the complex formation was −6.4 kcal mol^−1^, which corresponds to a dissociation constant of 4.94 × 10^4^ M^−1^ (Table 3).

Further, the analysis revealed that the 5-HT_1A_–**6** complex was stabilized mainly by many hydrogen bonds and hydrophobic interactions, including one hydrogen bond with SER^199^ and five hydrophobic interactions with VAL^117^ (Pi–Sigma and Pi–Alkyl), CYS^120^ (Pi–Alkyl), ILE^189^ (Pi–Alkyl), and PHE^361^ (Pi–Pi T-shaped) (Figure 6B). In addition, compound **6** formed van der Waals interactions with ASP^116^, THR^121^, ILE^124^, THR^196^, THR^200^, ALA^203^, PHE^362^, and ALA^365^. The binding energy and dissociation constant for the compound **6**–5-HT_1A_ interaction were −6.4 kcal mol^−1^ and 4.94 × 10^4^ M^−1^, respectively (Table 3).

The analysis of molecular docking also suggests that the 5HT_1A_–**7** complex was stabilized by hydrophobic interactions, including two Pi–Sigma hydrophobic interactions with VAL^117^, one Pi–Alkyl interaction with ILE^189^, one Pi–Sulfur bond with CYS^120^, and one Pi–Pi T-shaped bond with PHE^361^ (Figure 6C). In addition, it formed van der Waals interactions with ASP^116^, THR^121^, ILE^124^, TRY^195^, SER^199^, ALA^203^, and PHE^362^. Gibb’s free energy of the complex formation was −6.5 kcal mol^−1^, which corresponds to a dissociation constant of 5.85 × 10^4^ M^−1^ (Table 3).

Finally, Compound **8** was able to bind at the central binding cavity of 5-HT_1A_, and the resulting complex was mainly stabilized by hydrophobic interactions (Figure 6D). Compound **8** formed one hydrogen bond with THR^196^ and six hydrophobic (Pi–Alkyl) interactions with ILE^189^, LYS^191^, ALA^365^, and PRO^369^ (Figure 6D). In addition, it formed van der Waals interactions with VAL^117^, SER^190^, TYR^195^, SER^199^, THR^200^, PHE^361^, and PHE^362^. The binding energy and dissociation constant for compound **8** and 5-HT_1A_ interactions were −6.9 kcal mol^−1^ and 1.15 × 10^5^ M^−1^, respectively (Table 3).

##### 5-HT_2A_ Binding Interaction

The molecular docking of risperidone showed that it occupied the active site of 5-HT_2A_ and interacted primarily through hydrophobic interactions (Figure 7A,B). It formed two hydrogen bonds with SER^131^ and SER^159^ and a halogen bond with ASN^363^. Also, it formed two Pi–Sigma hydrophobic interactions with TRP^336^ and VAL^366^, one Pi–Pi T-shaped hydrophobic interaction with PHE^340^, two Alkyl hydrophobic interactions with ILE^163^ and VAL^366^, and seven Pi–Alkyl hydrophobic interactions with VAL^156^, PHE^243^, PHE^332^, TRP^336^, PHE^339^, PHE^340^, and VAL^366^ (Figure 7C). In addition, the protein–ligand complex was stabilized by van der Waals interactions with TYR^139^, TRP^151^, ASP^155^, THR^160^, LEU^228^, GLY^238^, SER^242^, and TYR^370^. The 5-HT_2A_–risperidone complex was stabilized by −11.8 kcal mol^−1^ free energy, which corresponds to a dissociation constant of 4.52 × 10^8^ M^−1^ (Table 4).

The analysis of molecular docking suggests that compound **5** occupied the active site of 5-HT_2A_ and the formed complex was stabilized by two hydrogen bonds with SER^159^ and THR^160^, and six hydrophobic interactions with TRP^336^, PHE^340^, VAL^156^, and ILE^163^ (Figure 8A). In addition, compound **5** formed van der Waals interactions with ASP^155^, SER^242^, PHE^243^, PHE^332^, PHE^339^, and TYR^370^. Gibb’s free energy of the complex formation was −7.3 kcal mol^−1^, which corresponds to a dissociation constant of 2.60 × 10^5^ M^−1^ (Table 4).

Moreover, the analysis revealed that the 5-HT_2A_–**6** complex was stabilized mainly by hydrogen bonds and hydrophobic interactions. Compound **6** formed one hydrogen bond with SER^242^ and eight hydrophobic interactions with TRP^336^, PHE^340^, SER^159^, THR^160^, VAL^156^, and ILE^163^ (Figure 8B). In addition, compound **6** formed van der Waals interactions with ASP^155^, THR^160^, GLY^238^, PHE^243^, PHE^332^, and PHE^339^. The binding energy and dissociation constant for compound 5-HT_2A_–**6** interactions were −7.5 kcal mol^−1^ and 3.17 × 10^5^ M^−1^, respectively (Table 4).

The analysis of molecular docking suggests that compound **7** occupied the active site of 5-HT_2A_, and the complex was stabilized via hydrogen bond formation and hydrophobic interactions. Compound **7** formed two hydrogen bonds with ASP^155^, four Pi–Pi T-shaped hydrophobic interactions with TRP^336^ and PHE^340^, one Pi–Alkyl interaction with VAL^156^, and two Amide–Pi stacked interactions with SER^159^ and THR^160^ (Figure 8C). In addition, compound **7** formed van der Waals interactions with LEU^123^, THR^160^, ILE^163^, GLY^238^, SER^242^, PHE^243^, PHE^332^, PHE^339^, and TYR^370^. Gibb’s free energy of the complex formation was −7.4 kcal mol^−1^, which corresponds to a dissociation constant of 2.68 × 10^5^ M^−1^ (Table 4).

Finally, the analysis of the 5-HT_2A_–**8** complex revealed that it was mainly stabilized by the formation of three hydrogen bonds with ASP^155^, THR^160^, and TYR^370^; three Pi–Pi T-shaped hydrophobic interactions with TRP^336^ and PHE^340^; and two Pi–Alkyl hydrophobic interactions with ILE^163^ and VAL^156^ (Figure 8D). A salt bridge was also formed between ASP^155^ and compound **8**. In addition, it formed van der Waals interactions with LEU^123^, SER^159^, SER^242^, PHE^243^, PHE^332^, PHE^339^, and VAL^366^. The binding energy and dissociation constant for 5-HT_2A_–**8** interactions were −8.1 kcal mol^−1^ and 2.68 × 10^5^ M^−1^, respectively (Table 4).

### 2.3. Prediction of Physicochemical, Pharmacokinetic, Drug-Likeness, and Toxicity

The physicochemical, pharmacokinetic (ADME parameters), drug-likeness, and toxicity properties of the investigated compounds were predicted using the SwissADME online tool [28]. These properties include lipophilicity, water-solubility, topological polar surface area (TPSA), GI absorption, blood–brain barrier (BBB) permeability, P-glycoprotein pump (P-gp) efflux, Lipinski’s rules, bioavailability, PAINS, and CYP^450^ inhibition. In general, the results showed compliance with Lipinski’s rules (Table 5).

The radar plot (Figure S9) is a representation of the mean values of six descriptors that are significant for oral bioavailability and used for a rapid appraisal of drug-likeness, including molecular size (SIZE), polarity (POLAR), lipophilicity (LIPO), flexibility (FLEX), saturation (SATU), and solubility (INSOLU). The red lines of the investigated molecules have to fall entirely in the pink area of the radar plot to be considered drug-like [28]. Most of the investigated compounds fall inside the pink area of their radar representations, indicating potential drug-like properties.

Lipophilicity is a crucial property for BBB permeability, a major obstacle in the delivery of antidepressant drugs to the brain [29]. Compounds **5**, **6**, and **7** showed potential BBB permeability; however, the permeability of the polar indole derivative (**8**) and the polyhydroxylated fatty acids (**3** and **4**) was not probable. The P-gp reduces the drug permeability through the BBB by allowing the efflux of many drugs back into the blood (multidrug resistance). No active efflux was observed by the P-gp for all investigated indole derivatives (Figure S10).

### 2.4. Analysis of Free Energy Calculations

The free energy (MM-GBSA) of the interaction between a protein and a ligand sheds light on the effect of solvent on the formation of a protein–ligand complex. Here, we calculated the free energy of the interaction of PPAR-γ with compounds **1**–**4**, and serotonin receptors (5-HT_1A_ and 5-HT_2A_) with compounds **5**–**8** (Table 6). It is clear that the free energies of compounds **1** (−54.55 kcal/mol) and **3** (−57.23 kcal/mol) were the lowest, suggesting that they formed a stable complex with PPAR-γ. Likewise, the free energies of compound **8** (−57.98 kcal/mol) for 5-HT_1A_ and compound **8** (−57.90 kcal/mol) for 5-HT_2A_ were the lowest, indicating that these compounds interacted favorably with 5-HT_1A_ and 5-HT_2A_, respectively. It is also imperative to note that van der Waals interactions (Δ*G*_vdW_), Coulombic interactions (Δ*G*_Coulomb_), and non-polar solvation energy (Δ*G*_SA_) or lipophilic interactions (Δ*G*_Sol_Lipo_) were the primary driving forces for the formation of a stable protein–ligand complex. On the other hand, polar solvation energy (Δ*G*_Solv_ or Δ*G*_SolGB_) and covalent (Δ*G*_Covalent_) interactions were the main forces to destabilize a protein–ligand complex. It is worth noting that we selected only compounds **1** and **3** for PPAR-γ, and compound **8** for 5-HT_1A_ as well as 5-HT_2A_ to gain an in-depth analysis of interaction by molecular dynamics (MD) simulation.

### 2.5. Analysis of Moelcular Dynamics Simulation (MDS)

#### 2.5.1. Root Mean Square Deviation (RMSD)

**Figure 9:**
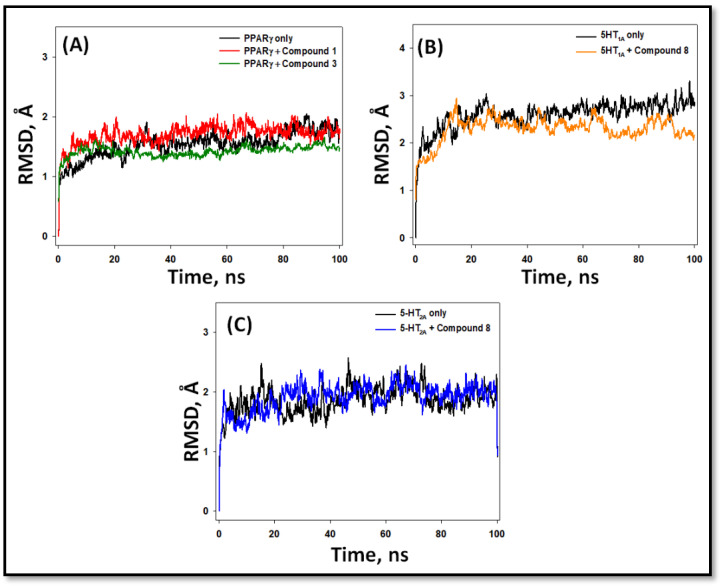
Root mean square deviation (RMSD) in the Cα-atoms of PPAR-γ, 5-HT_1A_, and 5-HT_2A_ with their respective compounds. (**A**) PPAR-γ alone and in the presence of compounds **1** and **3**; (**B**) 5-HT_1A_ alone and in the presence of compound **8**; (**C**) 5-HT_1A_ alone and in the presence of compound **8**.

RSMD is a measure of deviation in the structure of a protein in the presence or absence of a ligand from its initial structure during the course of simulation, which in turn reflects on the system’s stability [30]. In this study, RMSDs in Cα-atoms of PPAR-γ, 5-HT_1A_, and 5-HT_2A_ and their complexes, namely PPAR-γ-**1**, PPAR-γ-**3**, 5-HT_1A_-**8**, and 5-HT_2A_-**8** were determined (Figure 9). The RMSDs (between 20 and 100 ns) of PPAR-γ, 5-HT_1A_, and 5-HT_2A_ in the absence of any ligand fluctuated within 1.23–1.91 Å, 2.25–3.03 Å, and 1.39–2.57 Å, respectively, with average RMSDs of 1.79 ± 0.07 Å, 2.83 ± 0.10 Å, and 1.89 ± 0.06 Å, respectively. Further, the RMSDs in Cα-atoms of PPAR-γ (during 20–100 ns) in the presence of compounds **1** and **3** were within the range of 1.52–1.98 Å and 1.25–1.64 Å, respectively. The average RMSDs of PPAR-γ–**1** and PPAR-γ–**3** complexes were 1.82 ± 0.07 Å and 1.41 ± 0.04 Å, respectively (Figure 9A). Similarly, the RMSDs in Cα-atoms of 5-HT_1A_ and 5-HT_2A_ (during 20–100 ns) in the presence of compound **8** were within the range of 2.16–2.81 Å and 1.44–2.45 Å, respectively. The average RMSDs of 5-HT_1A_–**8** and 5-HT_2A_–**8** complexes were 2.44 ± 0.06 Å and 1.99 ± 0.05 Å, respectively (Figure 9B,C).

#### 2.5.2. Root Mean Square Fluctuation (RMSF)

**Figure 10:**
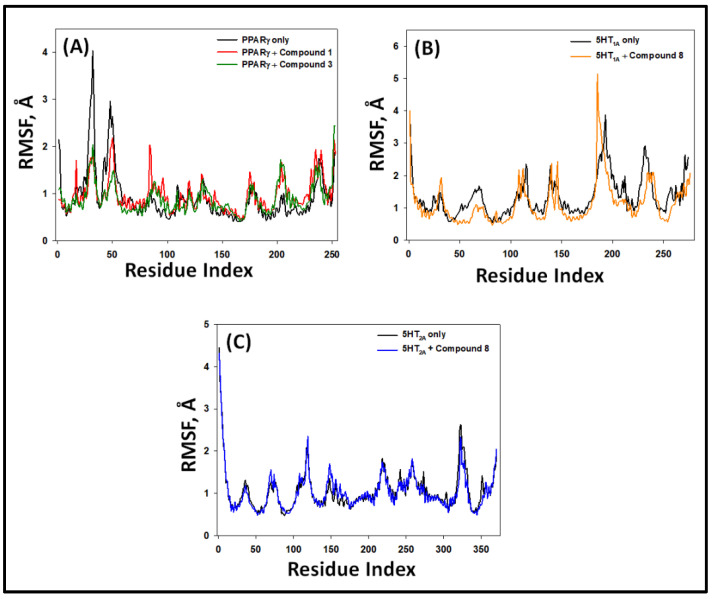
Root mean square fluctuation (RMSF) in the Cα-atoms of PPAR-γ, 5-HT_1A_, and 5-HT_2A_ with their respective compounds. (**A**) PPAR-γ alone and in the presence of compounds **1** and **3**; (**B**) 5-HT_1A_ alone and in the presence of compound **8**; (**C**) 5-HT_1A_ alone and in the presence of compound **8**.

During MD simulation, any fluctuations in the side chain of a protein due to the binding of a ligand are measured by monitoring the RMSF. Here, the RMSF values of PPAR-γ 5-HT_1A_ and 5-HT_2A_ alone or in the presence of their respective ligands were determined as a function of simulation time (Figure 10). The RMSF plot of PPAR-γ–**1** and PPAR-γ–**3** complexes overlapped with the RMSF plot of PPAR-γ alone, suggesting the absence of any significant changes in PPAR-γ conformation due to its interaction with compounds **1** and **3** (Figure 10A). Similarly, the RMSF plots of 5-HT_1A_–**8** and 5-HT_2A_–**8** complexes overlapped with the RMSF plots of 5-HT_1A_ and 5-HT_2A_ alone, respectively, showing that there were no significant changes in 5-HT_1A_ and 5-HT_2A_ due to the binding of ligands and hence the formation of stable protein–ligand complexes (Figure 10B,C). Any minor fluctuations in RMSF plots were due to the binding of ligands to proteins.

#### 2.5.3. Radius of Gyration (Rg)

**Figure 11:**
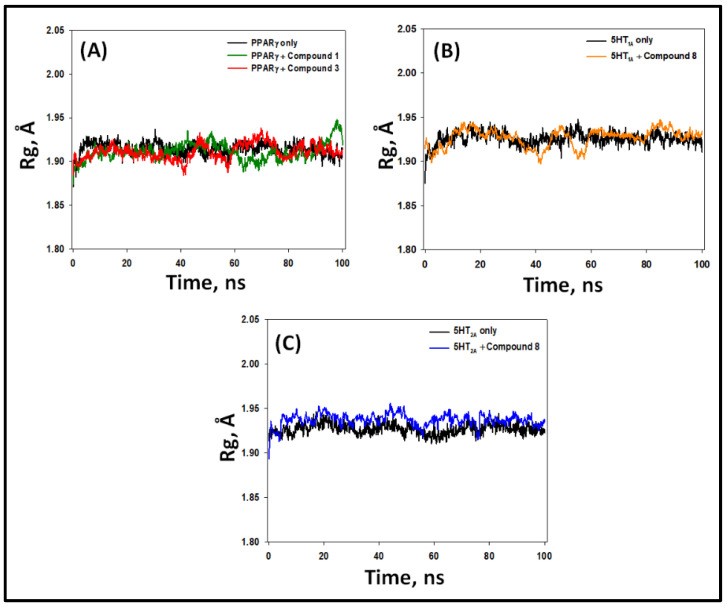
Variation in radius of gyration (Rg) of PPAR-γ, 5-HT_1A_, and 5-HT_2A_ with their respective compounds. (**A**) PPAR-γ alone and in the presence of compounds **1** and **3**; (**B**) 5-HT_1A_ alone and in the presence of compound **8**; (**C**) 5-HT_2A_ alone and in the presence of compound **8**.

The compactness of a protein–ligand complex, and hence its stability, are often measured by observing variation in Rg as a function of simulation time. We determined the Rg of PPAR-γ, 5-HT_1A_, and 5-HT_2A_ alone and their complexes, namely PPAR-γ–**1**, PPAR-γ–**3**, 5-HT_1A_–**8**, and 5-HT_2A_–**8**, during 100 ns of simulation (Figure 11). During 20–100 ns, the Rg values of PPAR-γ, 5-HT_1A_, and 5-HT_2A_ alone varied within 1.90–1.94 Å, 1.91–1.94 Å, and 1.92–1.95 Å, with average values of 1.93 ± 0.03 Å, 1.94 ± 0.05 Å, and 1.94 ± 0.06 Å, respectively. The Rg values of PPAR-γ–**1** and PPAR-γ–**3** complexes during 20–100 ns fluctuated within 1.88–1.93 Å and 1.89–1.94 Å, with an average value of 1.92 ± 0.07 Å and 1.93 ± 0.07 Å, respectively (Figure 11A). Similarly, the Rg values of 5-HT_1A_–**8** and 5-HT_2A_–**8** complexes during 20–100 ns fluctuated within 1.90–1.94 Å and 1.92–1.96 Å, with average values of 1.94 ± 0.06 Å and 1.94 ± 0.07 Å, respectively (Figure 11B,C). These results clearly signify that the compounds remain settled within the binding pocket of their respective proteins and form a stable protein–ligand complex.

#### 2.5.4. Solvent-Accessible Surface Area (SASA)

**Figure 12:**
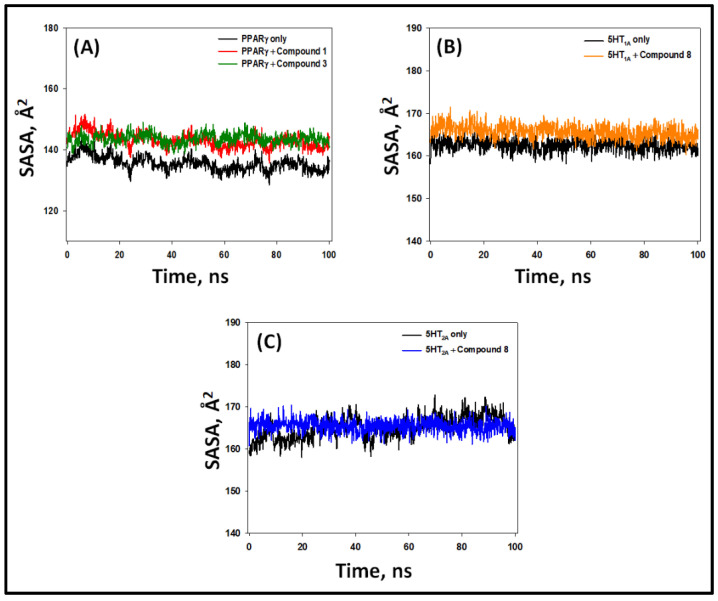
Variation in solvent-accessible surface area (SASA) of PPAR-γ, 5-HT_1A_, and 5-HT_2A_ with their respective compounds. (**A**) PPAR-γ alone and in the presence of compounds **1** and **3**, (**B**) 5-HT_1A_ alone and in the presence of compound **8**, (**C**) 5-HT_2A_ alone and in the presence of compound **8**.

SASA is a measure of the exposure of protein–ligand complexes to their surrounding solvent molecules, which in turn indicates the stability of a protein–ligand complex. Here, we determined the SASA of PPAR-γ, 5-HT_1A_, and 5-HT_2A_ alone and their complexes, namely PPAR-γ–**1**, PPAR-γ–**3**, 5-HT_1A_–**8,** and 5-HT_2A_–**8** (Figure 12). During 20–100 ns, the SASA values of PPAR-γ, 5-HT_1A_, and 5-HT_2A_ alone varied within 132–141 Å^2^, 159–165 Å^2^, and 157–172 Å^2^, with average values of 138 ± 4.3 Å^2^, 162 ± 5.1 Å^2^, and 165 ± 4.8 Å^2^, respectively (Figure 12). The SASA values of PPAR-γ–**1** and PPAR-γ–**3** complexes during 20–100 ns fluctuated within 138–152 Å^2^ and 139–149 Å^2^, with an average value of 143 ± 5.2 Å^2^ and 144 ± 7.1 Å^2^, respectively (Figure 12A). Similarly, the SASA values of 5-HT_1A_–**8** and 5-HT_2A_–**8** complexes during 20–100 ns fluctuated within 161–170 Å^2^ and 160–171 Å^2^, with an average value of 166 ± 6.3 Å^2^ and 167 ± 5.7 Å^2^, respectively (Figure 12B,C). These results clearly signify that the compounds remain seated within the binding pocket of their respective proteins and form a stable protein–ligand complex.

### 2.6. Principal Component Analysis (PCA) or Essential Dynamics (ED) Analysis

**Figure 13:**
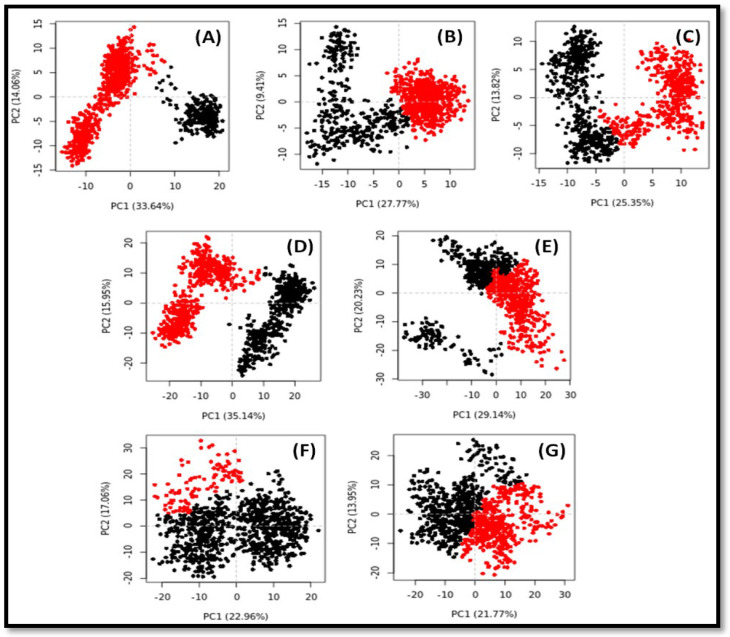
Principal component analysis (PCA) of PPAR-γ, 5-HT_1A_, and 5-HT_2A_ in the absence and presence of their respective ligands. (**A**) PPAR-γ alone; (**B**) PPAR-γ in the presence of compound **1**; (**C**) PPAR-γ in the presence of compound **3**; (**D**) 5-HT_1A_ alone; (**E**) 5-HT_1A_ in the presence of compound **8**; (**F**) 5-HT_2A_ alone; and (**G**) 5-HT_2A_ in the presence of compound **8**.

The global motion of a protein in the presence or absence of a ligand is generally monitored by PCA or ED [31]. In this study, the conformational sampling of Cα-atoms along PC1 and PC2 of PPAR-γ, 5-HT_1A_, and 5-HT_2A_ was performed in the absence or presence of their respective compounds (Figure 13). A conformational state of a protein is represented by the red and black dots. On the other hand, each red and black cluster shows the presence of distinct energetically favorable conformational spaces.

The conformational subspace occupied by PPAR-γ alone spans −15 to +20 along PC1 (33.64%), and −15 to +15 along PC2 (14.06%). Further, the conformational spaces occupied by PPAR-γ in the presence of compounds **1** and **3** were in the range of −18 to +15 along PC1 (27.77%)/−12 to +15 along PC2 (9.41%), and −15 to +15 along PC1 (25.35%)/−12 to +12 along PC2 (13.82%) respectively (Figure 13A–C). It is noticeable that the first three eigenvalues of PPAR-γ alone or in the presence of compounds **1** and **3** occupied 55.0%, 45.5%, and 48.2% conformational variances, respectively. Similarly, the conformational subspace occupied by 5-HT_1A_ alone spans −25 to +25 along PC1 (35.14%) and −15 to +22 along PC2 (15.95%). Further, the conformational space occupied by 5-HT_1A_ in the presence of compound **8** was in the range of −20 to +20 along PC1 (22.19%)/−30 to +20 along PC2 (18.51%) (Figure 13D,E). Similarly, the conformational space occupied by 5-HT_2A_ in the presence of compound **8** was in the range of −22 to +20 along PC1 (22.96%)/−20 to +30 along PC2 (17.06%) (Figure 13F,G).

Furthermore, the first three eigenvalues of 5-HT_1A_ alone, 5-HT_2A_ alone, 5-HT_1A_–**8** complex, and 5-HT_2A_–**8** complex occupied 58.1%, 49.4%, 56.4%, and 46.1% conformational variances, respectively. These results indicate that there was a marginal increase in the flexibility of 5-HT_1A_ in the presence of compound **8**, while the flexibility of PPAR-γ in the presence of compounds **1** and **3**, and flexibility of 5-HT_2A_ in the presence of compound **8** were similar to those of PPARγ alone and 5-HT_2A_ alone, respectively.

### 2.7. HPTLC Analysis of α-Linolenic in the Aerial Parts of S. irio

The developed HPTLC method was found to furnish a compact spot for α-linolenic acid at Rf = 0.57 ± 0.004 (Figure S11A). The regression equation/correlation coefficient (r^2^) for α-linolenic acid was Y = 6.49X + 2310.8/0.9971 in the linearity range of 100–1200 ng/band. The limits of detection (28.89 ng/band), quantification (87.57 ng/band), and recovery (98.16–99.26%) were found satisfactory for α-linolenic acid. The intra-/inter-day precisions (% RSD) for the proposed method were 1.24–1.48/1.14–1.43, which indicated a good precision for the proposed method. The amount of α-linolenic acid was estimated by comparing the peak area of the standard with that of crude extract (Figure S11B,C). Figure S11D clearly reveals that all peaks of α-linolenic acid in the extract coincided with each other at the observed UV absorption maxima (λ_max_ = 540). The estimated α-linolenic acid content in the hexane extract of aerial parts of *S. irio* was 28.67 μg/mg of dried extract.

## 3. Discussion

The chosen target for molecular docking analysis of the identified fatty acids was inspired by the activities reported in the literature for polyunsaturated fatty acids (PUFAs). PUFAs are known to reduce the risk of heart disease and heart attacks by refining blood lipids and endothelial function and by employing notable anti-inflammatory and anti-thrombotic effects [32]. They have a significant role in the treatment of type 2 diabetes through modulation of lipid and glucose homeostasis. They also play a vital role in Alzheimer’s disease and in some cancers [33].

PPAR-γ or PPARG is the peroxisome proliferator-activated receptor gamma, also known as the glitazone reverse insulin resistance receptor. It is a type II protein-regulating gene encoded by the PPAR-γ gene [34]. Polyunsaturated fatty acids (PUFAs) are known to function as agonists of PPAR-γ, a nuclear receptor that has been getting increasing interest as a novel therapeutic target for the treatment of diabetes and related metabolic disorders [35]. Studies demonstrated that activation of PPAR-γ by PUFA ligands results in a number of biologically beneficial effects, including stimulation of lipid and glucose metabolisms, anti-inflammatory effects, and favorable cardiovascular effects [36].

The results of docking of the fatty acids (**1**–**4**) revealed moderate interaction with PPAR-γ active residues that formed stable complexes with relatively high free energy, compared to the standard drug rivoglitazone. Compound **3** formed the most stable complex with the highest binding affinity (−7.4 kcal mol^−1^). On the other hand, the close structural similarity of indole alkaloids, some of which are of plant origin (exogenous agonists), to the endogenous neurotransmitter serotonin might explain the potential neurological activity of these compounds, as depression is mostly triggered by an imbalance in serotonin levels [37].

Assessment of the ADME properties of the isolated compounds revealed that all investigated compounds, except the polyunsaturated fatty acids **1** and **2**, showed high predicted GIT absorption and oral bioavailability. Regarding metabolism, CYP450–1A2 showed possible inhibition by compounds **5**, **6**, and **7**, whereas CYP2D6 showed potential inhibition by compounds **3** and **4**, in contrast to **8**, which showed no inhibition to all CYP450 subtypes. P-gp is extensively distributed in the capillary endothelial cells of the BBB and contributes to pumping xenobiotics back into the blood [38]. Bypassing the P-gp drug-efflux mechanism is a crucial property for drugs used in neurodegenerative diseases [38,39]. In other words, compounds that are not P-gp substrates are predictably more bioavailable in the brain [28]. Since no predictable active efflux was observed by the P-gp for the investigated indole derivatives (**5**–**8**), they can be delivered in appropriate concentrations to the brain and used in the treatment of neurological disorders, including depression [29], whereas **3** and **4** are probable P-gp substrates due to the presence of more rotatable bonds compared to indole derivatives (**5**–**8**) [40]. Also, no PAINS (Pan-assay interference compounds) alerts were detected for any of the tested compounds (**1**–**8**).

A study conducted on the extract of *Mitragyna speciosa*, which contains the indole alkaloids mitragynine, paynantheine, and speciociliatine as major constituents, induced an antidepressant-like effect in mouse models; the effect was speculated to be through the interaction with the hypothalamic–pituitary–adrenal (HPA) axis in the neuroendocrine system [41].

Another plant, *Passiflora incarnata* L. (passion flower), containing the indole alkaloids harman, harmol, and harmine, reduced anxiety and improved memory in rats in a dose-dependent manner. Cortical serotonin content was depleted, with increased levels of metabolites and increased turnover. It was found that the proposed mechanism of action of passion flower involved GABAA-receptors [42]. These facts motivated us to investigate the potential neurological activity of currently isolated indole alkaloids through docking into the 5-HT_1A_ and 5-HT_2A_ receptors. The serotonin receptor subtype 5-HT_1A_ has been implicated in several neurological conditions, and 5-HT_1A_ receptor agonism represents efficacious therapeutic potential for the treatment of major depression, anxiety, schizophrenia, and Parkinson’s disease.

## 4. Materials and Methods

### 4.1. Apparatus and Chemicals

IR spectrum was acquired using a JASCO 320-A spectrometer (JASCO International Co., Ltd., Easton, MD, USA). Normal and reversed-phase silica gels (Merck, Darmstadt, Germany) were used for column chromatography (CC) and thin-layer chromatography (TLC). The compounds were visualized on TLC by spraying with 15% H_2_SO_4_/ethanol, followed by heating.

NMR spectroscopy was performed using deuterated solvents in an UltraShield Plus 500 (Bruker, Billerica, MA, USA) spectrometer operating at 500 MHz for ^1^H and 125 MHz for ^13^C at the College of Pharmacy, Prince Sattam Bin Abdulaziz University. The two-dimensional NMR analyses (COSY, HSQC, and HMBC) were conducted using the standard Bruker pulse program. Chemical shift values are reported in δ (ppm) relative to an internal standard (TMS), and coupling constants (J) are reported in Hertz (Hz).

HRMS was performed using a Thermo Scientific UPLC RS Ultimate 3000 Q Exactive Hybrid Quadrupole-Orbitrap Mass Spectrometer (Mundelein, IL, USA) combined with high-performance quadrupole precursor selection with high resolution, accurate-mass (HR/AM) Orbitrap™ detection. The instrument was located at Prince Sattam Bin Abdulaziz University, College of Pharmacy. The detection was performed in negative and positive modes, and the experiment run time was 1 min using nitrogen as the supplementary gas with a scan range from 160–1500 m/z.

### 4.2. Plant Material

The aerial parts of *S. irio* L. were collected from a farm near Riyadh city in the Najd region of Saudi Arabia in March 2019 and kindly identified by a taxonomist at the Pharmacognosy Department, College of Pharmacy, King Saud University. A voucher specimen (no. 14380) has been deposited in the herbarium of the Pharmacognosy Department, College of Pharmacy.

### 4.3. Extraction and Isolation of Compounds

The shade-dried aerial parts of the plant (1 kg) were coarsely powdered and extracted with 80% ethanol. The ethanolic extract was concentrated under reduced pressure using a rotary evaporator (R-210, BUCHI) to give 28 g of brownish-black mass. The obtained extract was fractionated using different polarity solvents, starting with *n*-hexane (*n*-Hex.), followed by dichloromethane (CH_2_Cl_2_), and finally *n*-butanol (*n*-BuOH) to obtain the corresponding fractions.

The *n*-Hex. fraction (8 g) was chromatographed over a silica gel column, starting with CHCl_3_ as a mobile system, and gradually increasing polarity with MeOH. The eluted fractions were monitored with TLC, and similar fractions were combined to end up with 16 main fractions (*n*-Hex.1–16). Fraction *n*-Hex.2, eluted with 2% MeOH/CHCl_3_, was purified using chromatotron (CPTL, silica gel 60 GF_254_, 1 mm), 5% EtOAc/*n*-Hex. as mobile phase, to obtain compound **1** in pure form. Another part of the *n*-Hex.2 fraction was subjected to RP-18 column chromatography (CC) using 5% H_2_O/CH_3_CN as a solvent system to provide compounds **2** and **3** in pure forms.

Fraction *n*-Hex.7, eluted with 5% MeOH/CHCl_3_, was subjected to Rp-18 CC using 5% H_2_O/MeOH as a solvent system to give compound **4**. Fraction *n*-Hex.16, eluted with 20% MeOH/CHCl_3_, was purified by using centrifugal thin-layer chromatography (mobile phase: 0.5% MeOH/EtOAc) to provide compound **5**.

Part of the CH_2_Cl_2_ fraction (6 g) was purified using a chromatotron (1 mm, mobile phase, 4% MeOH/CHCl_3_) to obtain several subfractions (1–28); further purification of sub-fraction 17 resulted in compound **6** in pure form.

Part of the *n*-BuOH fraction (5 g) was loaded on top of a silica gel column and eluted with a gradient solvent system of *n*-BuOH–water–acetic acid 13:2:1 *v*/*v*/*v* to produce five subfractions (*n*-BuOH.1–5). Sub-fractions *n*-BuOH.1 and *n*-BuOH.2, were purified by Rp-18 CC using 10% CH_3_CN/H_2_O to yield compounds **7** and **8**, respectively.

### 4.4. Molecular Docking

Interaction of the active constituents of *S. irio* with serotonin receptors (5-HT_1A_ and 5-HT_2A_) and PPAR-γ was studied by performing molecular docking using AutoDock 4.2 [43,44]. The two-dimensional structures of ligands (active constituents) were drawn in ChemDraw Ultra 7.0 and converted to three-dimensional structures using OpenBabel. In ligands, Gasteiger partial charges were added, non-hydrogen atoms were merged, and rotatable bonds were defined using AutoDock Tools (ADT). The energies of all the ligands were minimized using the Universal Forcefield (UFF). The three-dimensional coordinates of different drug targets were obtained from the Protein Data Bank (www.rcsb.org, accessed on 21 August 2022). The X-ray crystal structure (PDB ID: 5U5L) of PPAR-γ in complex with rivoglitazone was resolved to 2.55 Å [10]. Similarly, the X-ray crystal structures of both serotonin receptors, namely 5-HT_1A_ (PDB ID: 7E2Y) and 5-HT_2A_ (PDB ID: 6A93), bound with serotonin and risperidone, respectively, were resolved to 3.00 Å [45,46].

Prior to molecular docking, the target proteins were cleaned by removing any heteroatoms, including non-essential water molecules, and adding hydrogen atoms. Also, Kollman-united atom type charges and solvation parameters were added with the help of ADT. For PPAR-γ, grid boxes were defined as 35Å × 35Å × 35Å centered at −5 Å, 33 Å, and 131 Å coordinates. Similarly, the dimensions of grid boxes of 5-HT_1A_ and 5-HT_2A_ were set at 28Å × 28Å × 28Å placed at 101 Å, 115 Å, and 108 Å; and 35Å × 28Å × 29Å centered at 16 Å, −0.2Å, and 60Å, respectively. Molecular docking was performed using the Lamarck Genetic Algorithm (LGA) along with the Solis and Wets search methods. The position, torsion, and orientation of ligands were set randomly, and all rotatable torsions were released. For each docking run, a maximum of 2.5 × 10^6^ energy calculations were computed. The population size, translational step, quaternions, and torsion steps were set at 150, 0.2, 5, and 5, respectively. For each docking experiment, the lowest-energy docked structure was selected from 10 runs. Discovery Studio Visualizer was used to prepare and analyze the results and prepare figures. The dissociation constant (*K*_d_) was evaluated from binding free energies (Δ*G*) using the following equation.

$$\Delta G=-RTlnK_{d}$$

where *R* and *T* were the universal gas constant (=1.987 cal/mol/K) and temperature (=298K), respectively.

### 4.5. Prediction of Physicochemical, Pharmacokinetic, Drug-Likeness, and Toxicity

The physicochemical, pharmacokinetic, drug-likeness, and toxicity properties of the investigated compounds were predicted using the SwissADME web tool hosted by the Swiss Institute of Bioinformatics (http://www.sib.swiss, accessed on 24 August 2022) [28].

### 4.6. Molecular Dynamics Simulation (MDS)

The MDS of PPARγ, 5-HT_1A_, and 5-HT_2A_ along with their respective ligands (compounds **1**, **3**, **7**, and **8**) was performed using Desmond-2018 (Schrodinger, LLC, NY, USA), as described earlier [47,48]. The MDS was performed in an orthorhombic box by placing the initial protein–ligand docked pose at the center of the box, keeping a distance of at least 10 Å from the box boundaries. The simulation box was solvated with TIP3P water molecules, and Na^+^ or Cl^−^ ions were added to neutralize the system. Salt (150 mM NaCl) was added to the system to mimic the physiological condition. The system was iterated with 1000 steps with a convergence criterion of 1 kcal/mol/Å using an OPLS3e force field in order to minimize its energy. A 100 ns production run was initiated using the OPLS3e force field under NPT conditions of 298 K temperature and 1 bar pressure. A Nose–Hoover chain thermostat and Martyna–Tobias–Klein barostat were employed to maintain the NPT conditions of the system, respectively [49,50]. A time step of 2 fs was kept in all MDS, and at every 10 ps, energies and structures were saved in the trajectory. The trajectories were analyzed for root mean square deviation (RMSD), root mean square fluctuation (RMSF), radius of gyration (Rg), and solvent-accessible surface area (SASA).

### 4.7. Free Energy Calculations

The free energy of protein–ligand complex formation was computed by the MM–GBSA (Molecular Mechanics–Generalized Born Surface Area) approach using the Prime-2018 module (Schrodinger, LLC, New York, NY, USA), as described previously [51]. Briefly, the Molecular Mechanics (MM) approach was first used to locally optimize the docked complexes, and then their energies were minimized employing an OPLS3e force field along with a generalized Born surface area (GBSA) continuum solvent. The following relations were utilized to calculate the binding free energies of protein–ligand complexes:

$$\Delta G_{Bind}=\Delta E_{MM}+\Delta G_{Solv\_GB}+\Delta G_{SA}$$

$$\Delta E_{MM}=E_{complex}-\left(E_{protein}+E_{ligand}\right)$$

where *E_complex_*, *E_protein_*, and *E_ligand_* are the minimized energies of the protein–ligand complex, the protein alone, and the ligand alone, respectively;

$$\Delta G_{solv\_GB}=G_{solv_{GB}(complex)}-G_{solv_{GB\left(protein\right)}}+G_{solv\_GB(ligand)}$$

where *G_solv_GB(complex)_*, *G_solv_GB(protein)_*, and *G_solv_GB_*_(*ligand)*_ are the free energies of solvation of the protein–ligand complex, the protein alone, and the ligand alone, respectively; and

$$\Delta G_{SA}=G_{SA(compelx)}-G_{SA\left(protein\right)}+G_{SA(ligand)}$$

where *G_SA(complex)_*, *G_SA(protein)_*, and *G_SA(ligand)_* are the surface area energies of the protein–ligand complex, the protein alone, and the ligand alone, respectively.

In the Prime-MM/GBSA method, the free energy is calculated as follows:

$$\Delta G_{Bind}=\Delta G_{Coulomb}+\Delta G_{vdW}+\Delta G_{Covalent}+\Delta G_{H-bond}+\Delta G_{Sol\_Lipo}+\Delta G_{Solv\_GB}+\Delta G_{Packing}+\Delta G_{Self-contact}$$

### 4.8. Principal Component Analysis (PCA) or Essential Dynamics (ED)

The collective motions of proteins along with their respective ligands were measured by employing a PCA or essential dynamics (ED) approach using the Bio3D package [52,53]. In this approach, first the protein’s translational and rotational motions are disregarded, followed by the calculation covariance matrix and its eigenvectors by superimposing the protein’s atomic coordinates onto a reference structure. Secondly, the symmetric matrix is diagonalized by an orthogonal transformation matrix, giving a diagonalized matrix of eigenvalues. The covariance matrix (*C*) is calculated using the following relation:

$$C_{ij}=\langle \left(x_{i}-\langle x_{i}\rangle \right)\left(x_{j}-\langle x_{j}\rangle \right)\rangle \;\;\;\;\;\;i,j=1,2,3,\ldots \ldots ,3N$$

where, *N*, x*_i_*_/*j*_, and <*x_i/j_*> represent the number of Cα-atoms, the Cartesian coordinates of the *i*^th^/*j*^th^ Cα-atom, and time average of all the conformations, respectively.

### 4.9. Standardization of S. irio Extract by a Validated HPTLC Method

The standardization of *S. irio* extract was carried out by a validated high performance thin layer chromatography (HPTLC) method using α-linolenic acid as the marker compound. Chromatography was performed on a glass-backed silica gel 60 F_254_ HPTLC plate (20 × 10 cm). Different combinations of solvents were tested to develop the HPTLC method, and a mixture of acetone, *n*-hexane, and acetic acid in the proportion of 25:75:0.1 *v*/*v*/*v* was selected as the most suitable mobile phase. Application of α-linolenic acid and the extracts on chromatographic plates (band wise) was carried out by an automatic TLC sampler-4 (ATS-4) while the development of the plate took place in ADC-2 (Automatic Development Chamber-2). Post development, the plate was derivatized with vanillin sulfuric acid reagent and heated to give compact bands of the chosen marker compound. It was scanned and quantified densitometrically at λ_max_ = 540 nm. The developed method was validated for precision, recovery, robustness, limits of detection (LOD), and limits of quantification (LOQ) in accordance with ICH guidelines.

## 5. Conclusions

Chromatographic investigation of the aerial parts of the edible plant *S. irio* resulted in the isolation of eight compounds, of which four (**1**–**4**) are unsaturated fatty acids and the other four (**5**–**8**) are identified as indole alkaloids. The structure of compound 4 was established as the fatty acid 8,11,12-trihydroxy-9*Z*,15*Z*-octadecadienoic acid, which is reported here for the first time from a natural source. Different spectroscopic techniques such as 1D, 2D NMR, and MS were employed to confirm the identity of the isolated compounds. Further, in silico molecular docking studies of compounds 1–4 were performed against PPAR-γ, which confirmed the agonist activity of compound **3** with a binding energy of −7.4 kcal mol^−1^ compared to the antidiabetic drug rivoglitazone. Similarly, molecular docking studies of compounds **5**–**8** were performed against serotonin receptor subtypes, namely 5-HT_1A_ and 5-HT_2A_. Compound 8 exhibited notable docking scores, suggesting the strongest affinity among the tested indoles, with binding energies of −6.9 kcal/mol to 5HT_1A_ and −8.1 kcal/mol to 5HT_2A_, respectively, against serotonin and risperidone as positive controls. The stability of target protein and compound complexes was tested by performing molecular dynamics simulations and analyzing parameters such as RMSD, RMSF, Rg, and SASA, which confirmed the formation of stable protein complexes. Further, principal component analysis (PCA) was used to collectively monitor the motion of amino acid residues of target proteins (PPAR-γ, 5-HT_1A_, and 5-HT_2A_) in the presence of their respective compounds. In addition, an HPTLC method was developed for the quantification of the biomarker compound **2**, which guarantees its application in quality control of commercialized herbal drugs and formulations containing α-linolenic acid. This study’s outcome may serve as a scaffold to construct novel derivatives with higher potency and desirable drug-like properties. However, further validations through in vitro and in vivo studies are required.

## Figures and Tables

**Table 1:** ^1^H (500 MHz) and ^13^C NMR (125 MHz) data (in CD_3_OD) for compound **4**.

No.	4	
	δ_C_, Mult.	δ_H_, Mult (*J* in Hz)
1	177.9, C	-
2	35.0, CH_2_	2.16, t (7.3)
3	26.0, CH_2_	1.48, br t (7.2)
4	30.1, CH_2_	1.22, m
5	30.3, CH_2_	1.22, m
6	26.4, CH_2_	1.22, m
7	38.2, CH_2_	1.41, m
8	73.0, CH	3.98, m
9	136.5, CH	5.60, m
10	131.0, CH	5.60, m
11	75.8, CH	3.85, t (6.0)
12	75.7, CH	3.38, m
13	31.5, CH_2_	a. 2.24, m b. 2.02, m
14	30.5, CH_2_	1.22, m
15	126.3, CH	5.34, m
16	134.4, CH	5.34, m
17	21.6, CH_2_	1.95 br. t (6.5)
18	14.6, CH_3_	0.85, t (7.4)

**Table 2:** Molecular docking parameters for the interaction of isolated SI fatty acids (**1**–**4**) with PPAR-γ.

Compound	Δ*G* kcal mol^−1^	Receptor Amino Acids
Rivoglitazone	−8.2	ARG^280^, ILE^281^, PHE^282^, GLY^284^, CYS^285^, GLN^286^, ARG^288^, SER^289^, HIS^323^, ILE^326^, TYR^327^, LEU^330^, VAL^339^, ILE^341^, MET^348^, LEU^353^, MET^364^, HIS^449^, LEU^453^, LEU^469^, TYR^473^
**1**	−6.7	PHE^226^, CYS^285^, GLN^286^, ARG^288^, SER^289^, ALA^292^, GLU^295^, ILE^296^, HIS^323^, ILE^325^, ILE^326^, TYR^327^, MET^329^, LEU^330^, LEU^333^, LYS^367^, HIS^449^, LEU^453^, LEU^465^, LEU^469^, TYR^473^
**2**	−6.0	LEU^228^, PHE^282^, CYS^285^, GLN^286^, ARG^288^, SER^289^, ALA^292^, HIS^323^, ILE^326^, TYR^327^, MET^329^, LEU^330^, SER^332^, LEU^333^, PHE^363^, MET^364^, HIS^449^, LEU^453^, LEU^465^, LEU^469^, TYR^473^
**3**	−7.4	LEU^228^, ILE^281^, PHE^282^, CYS^285^, GLN^286^, ARG^288^, SER^289^, ALA^292^, ILE^326^, TYR^327^, MET^329^, LEU^330^, SER^332^, LEU^333^, LEU^356^, PHE^360^, PHE^363^, MET^364^, LYS^367^, HIS^449^
**4**	−6.1	PHE^282^, CYS^285^, GLN^286^, ARG^288^, SER^289^, ALA^292^, HIS^323^, ILE^326^, TYR^327^, MET^329^, LEU^330^, LEU^333^, PHE^363^, MET^364^, LYS^367^, HIS^449^, LEU^453^, LEU^465^, LEU^469^, TYR^473^

Arg: Arginine; Ile: Isoleucine; Phe: Phenylalanine; Gly: Glycine; Cys: Cysteine; Gln: Glutamine; Ser: Serine; His: Histidine; Tyr: Tyrosine; Leu: Leucine; Val: Valine; Met: Methionine; Glu: Glutamic acid; Ala: Alanine.

**Table 3:** Molecular docking parameters for the interaction of isolated indole compounds (**5**–**8**) with 5-HT_1A_ serotonin receptor.

Compound	Δ*G* kcal mol^−1^	Receptor Amino Acids
Serotonin	−6.1	ASP^116^, VAL^117^, CYS^120^, THR^121^, ILE^124^, ILE^167^, ILE^189^, SER^199^, ALA^203^, PHE^361^, PHE^362^, ALA^365^, ASN^386^, TYR^390^
**5**	−6.4	ASP^116^, VAL^117^, CYS^120^, THR^121^, ILE^124^, ALA^203^, TRP^358^, PHE^361^, PHE^362^, ASN^386^, GLY^389^, TYR^390^
**6**	−6.4	ASP^116^, VAL^117^, CYS^120^, THR^121^, ILE^124^, ILE^189^, THR^196^, SER^199^, THR^200^, ALA^203^, PHE^361^, PHE^362^, ALA^365^
**7**	−6.5	ASP^116^, VAL^117^, CYS^120^, THR^121^, ILE^124^, ILE^189^, TRY^195^, SER^199^, ALA^203^, PHE^362^
**8**	−6.9	VAL^117^, ILE^189^, SER^190^, LYS^191^, TYR^195^, THR^196^, SER^199^, THR^200^, PHE^361^, PHE^362^, ALA^365^, PRO^369^

Ala: Alanine; Asn: Asparagine; Asp: Aspartic acid; Cys: Cysteine; Gly: Glycine; Ile: Isoleucine; Lys: Lysine; Phe: Phenylalanine; Pro: Proline; Ser: Serine; Thr: Threonine; Tyr: Tyrosine; Val: Valine.

**Table 4:** Molecular docking parameters for the interaction of isolated SI indole compounds (**5**–**8**) with 5-HT_2A_ serotonin receptor.

Compound	Δ*G* kcal mol^−1^	Receptor Amino Acids
Risperidone	−11.8	SER^131^, TYR^139^, TRP^151^, ASP^155^, VAL^156^, SER^159^, THR^160^, ILE^163^, LEU^228^, GLY^238^, SER^242^, PHE^243^, PHE^332^, TRP^336^, PHE^339^, PHE^340^, ASN^363^, VAL^366^, TYR^370^
**5**	−7.3	ASP^155^, VAL^156^, SER^159^, THR^160^, ILE^163^, TRP^336^, SER^242^, PHE^243^, PHE^332^, TRP^336^, PHE^339^, PHE^340^, TYR^370^
**6**	−7.5	ASP^155^, VAL^156^, SER^159^, THR^160^, ILE^163^, GLY^238^, SER^242^, PHE^243^, PHE^332^, TRP^336^, PHE^339^, PHE^340^
**7**	−7.4	LEU^123^, ASP^155^, VAL^156^, SER^159^, THR^160^, ILE^163^, GLY^238^, SER^242^, PHE^243^, PHE^332^, TRP^336^, PHE^339^, PHE^340^, TYR^370^
**8**	−8.1	LEU^123^, ASP^155^, VAL^156^, SER^159^, THR^160^, ILE^163^, SER^242^, PHE^243^, PHE^332^, TRP^336^, PHE^339^, PHE^340^, VAL^366^, TYR^370^

**Table 5:** The predictive physicochemical, pharmacokinetic, drug-likeness, and toxicity properties of isolated *SI* compounds (**1**–**8**).

Property	Compound ID							
	1	2	3	4	5	6	7	8
Molecular weight	250.38	278.43	330.46	328.44	186.2140	156.18	190.2020	203.2450
Molecular formula	C_16_H_26_O_2_	C_18_H_30_O_2_	C_18_H_34_O_5_	C_18_H_32_O_5_	C_11_H_10_N_2_O	C_11_H_10_N_2_O	C_10_H_10_N_2_O_2_	C_11_H_13_N_3_O
Lipophilicity (Log *P*_o/w_)	4.88	5.66	3.02	2.80	2.23	1.77	0.80	0.52
Water solubility (ESOL)	Insoluble	Insoluble	Soluble (−2.88)	Soluble (−2.88)	Soluble (−2.64)	Soluble (−2.31)	Soluble (−2.47)	Very soluble (−1.71)
TPSA	37.3	37.3	97.99	97.99	37.95	39.58	57.25	84.9
Lipinski	Yes	Yes	Yes	Yes	Yes	Yes	Yes	Yes
GIT absorption	---	---	High	High	High	High	High	High
BBB permeability	---	---	No	No	Yes	Yes	Yes	No
P-gp substrate	---	---	Yes	Yes	No	No	No	No
Bioavailability score	---	---	0.56	0.56	0.55	0.55	0.55	0.55
H-bond (donors/acceptors)	1/2	1/2	4/5	4/5	0/2	1/1	1/2	3/2
CYP450-1A2	---	---	No	No	Yes	Yes	Yes	No
CYP2D6	---	---	Yes	Yes	No	No	No	No
CYP2C19/2C9/3A4	---	---	No	No	No	No	No	No
PAINS	No	No	No	No	No	No	No	No

**Table 6:** Calculation of free energy (MM-GBSA) for the interactions between PPAR-γ and 5-HT_1A_ with the SI compounds (**1**–**8**).

Target Protein	Compounds	Δ*G* or Δ*G*_Bind_	Δ*G*_Coulomb_	Δ*G*_Covalent_	Δ*G*_H-bond_	Δ*G*_SA_ or Δ*G*_Sol_Lipo_	Δ*G*_Packing_	Δ*G*_Solv_ or Δ*G*_SolGB_	Δ*G*_vdW_
PPAR-γ	Compound **1**	−54.55	−46.20	10.06	−2.74	−8.54	−5.20	51.15	−53.08
	Compound **2**	−45.58	−18.22	6.91	−2.11	−15.70	−0.49	19.58	−35.55
	Compound **3**	−57.23	−52.92	9.92	−3.90	−11.13	−4.30	55.84	−50.74
	Compound **4**	−49.86	−31.73	2.89	−3.04	−8.09	−1.71	30.21	−38.39
5-HT_1A_	Compound **5**	−52.79	−11.85	5.18	−2.37	−17.41	−5.54	18.30	−39.10
	Compound **6**	−44.92	2.00	−0.49	−1.54	−10.24	−4.99	1.81	−31.47
	Compound **7**	−55.02	−18.20	2.84	−1.68	−17.12	−7.56	21.31	−34.61
	Compound **8**	−57.98	−9.78	4.95	−1.19	−19.13	−4.56	12.31	−40.58
5-HT_2A_	Compound **5**	−28.18	−23.58	17.14	−2.13	−13.08	−1.58	25.34	−30.29
	Compound **6**	−45.58	−18.22	6.91	−2.11	−15.70	−0.49	19.58	−35.55
	Compound **7**	−36.15	−17.54	0.83	−1.16	−8.45	−7.36	13.55	−16.02
	Compound **8**	−57.90	−9.78	4.95	−1.19	−19.13	−4.56	12.31	−40.5

All energies are in kcal mol^−1^. Δ*G*_Coulomb_, Δ*G*_vdW_, Δ*G*_Covalent_, Δ*G*_Solv_ or Δ*G*_SolGB_, Δ*G*_H-bond_, Δ*G*_SA_ or Δ*G*_Sol-Lipo_, Δ*G*_Packing_, and Δ*G* or Δ*G*_Bind_ stand for minimized molecular mechanics energy, Coulomb energy, van der Waals energy, covalent binding energy, solvation energy, energy due to self-contact, energy due to H-bonds, lipophilic energy, packing energy, and binding energy, respectively.

## Data Availability

Not applicable.

## Supplementary Materials

The following supporting information can be downloaded at: https://www.mdpi.com/article/10.3390/ph16040498/s1, Figure S1: Chemical structures of the isolated compounds (**1**–**8**), Figures S2–S8: 1D, 2D NMR, and HRESIMS spectra of compound **4**, Figure S9: Bioavailability radar representations of the isolated compounds (**1**–**8**), Figure S10: Boiled-egg graph of blood–brain barrier (BBB) permeability and human gastrointestinal absorption (HIA), glycoprotein substrate (PGP^+^), and non-substrate (PGP^–^), Figure S11: Chromatogram of HPTLC analysis of linolenic acid in hexane extract of *S. irio* (aerial parts); Table S1: ^1^H (500 MHz) and ^13^C (125 MHz) NMR data of compounds **1**, **2**, and **3**, Table S2: ^1^H (500 MHz) and ^13^C (125 MHz) NMR data of compounds **5**–**8**, Table S3: Binding parameters for the interaction of *S. irio* compounds **1**–**4** with PPAR-γ, Table S4: Binding parameters for the interaction of *S. irio* compounds **5**–**8** with the 5-HT_1A_ serotonin receptor, Table S5: Binding parameters for the interaction of *S. irio* compounds **5**–**8** with the 5-HT_2A_ serotonin receptor.
