# Supplementary material for: Biomarker Quantification, Spectroscopic, and Molecular Docking Studies of the Active Compounds Isolated from the Edible Plant Sisymbrium irio L
Source: Pharmaceuticals (Basel). 2023 Mar 27;16(4):498. doi: 10.3390/ph16040498 (PMC10146147; doi:10.3390/ph16040498)
Supplement: Supplementary file 1 [file pharmaceuticals-16-00498-s001.zip › pharmaceuticals-2206961-supplementary.pdf]

Biomarker quantification, spectroscopic, and molecular docking studies of the active compounds isolated from the edible plant *Sisymbrium irio* L.

Shaza M. Al-Massarani [1], Latifah S. Aldurayhim [1], Ibtisam A. Alotaibi [1], Mostafa W. M. Abdelmageed [1], Md Tabish Rehman [1], Omer A. Basudan [1], Maged S. Abdel-Kader [2,3], Mohamed F. Alajmi [1], Fatma M. Abdel Bar [2,4], Perwez Alam[1], Maram M. Al Tamimi [1] and Ali A. ElGamal [1,4,*]

[1] Department of Pharmacognosy, College of Pharmacy, King Saud University, P.O. Box 2457, Riyadh 11451, Saudi Arabia
[2] Pharmacognosy Department, College of Pharmacy, Sattam Bin Abdulaziz University, Al-kharj 11942, Saudi Arabia
[3] Department of Pharmacognosy, College of Pharmacy, Alexandria University, Alexandria 21215, Egypt
[4] Pharmacognosy Department, Faculty of Pharmacy, Mansoura University, El Mansoura 35516, Egypt

* Correspondence: *E-mail address*: aelgamel@ksu.edu.sa (Ali A. El-Gamal).

Abstract: Phytochemical investigation of the ethanolic extract of the aerial parts of *Sisymbrium irio* L. led to the isolation of four unsaturated fatty acids (**1-4**), including a new one (**4**), and four indole alkaloids (**5-8**). The structures of the isolated compounds were characterized with the help of spectroscopic techniques such as 1D, 2D NMR, mass spectroscopy and by correlation with the known com-pounds. In terms of their notable structural diversity, molecular docking approach with the AutoDock 4.2 program was used to analyze the interactions of the identified fatty acids with PPAR-γ and the indole alkaloids with 5-HT_{1A} and 5-HT_{2A}, subtypes of serotonin receptor, respectively. Compared to the antidiabetic drug rivoglitazone, compound **3** acted as a potential PPAR-γ agonist with binding energy -7.4 kcal mol-1. Moreover, compound **8** displayed the strongest affinity, with binding energies of -6.9 kcal/mol to $5HT_{1A}$, and -8.1 kcal/mol to $5HT_{2A}$, using serotonin and the antipsychotic drug risperidone as positive controls, respectively. The results of docked conformations represent an interesting target for developing novel antidiabetic and antipsychotic drugs and warrant further evaluation of these ligands in vitro and in vivo. On the other hand, an HPTLC method was developed to quantify α-linolenic acid, in the hexane fraction of the ethanol extract of *S. irio*. The regression equation/correlation coefficient (r2) for linolenic acid was found as Y= 6.49X + 2310.8/ 0.9971 in the linearity range of 100-1200 ng/band. The content of α-linolenic acid in *S. irio* aerial parts was found to be 28.67 µg/mg of dried extract.

Keywords: *Sisymbrium irio;* unsaturated fatty acids; indole alkaloids; molecular docking; HPTLC standardization

1

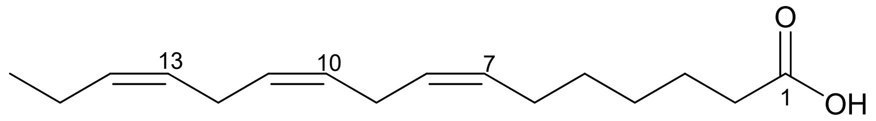

2

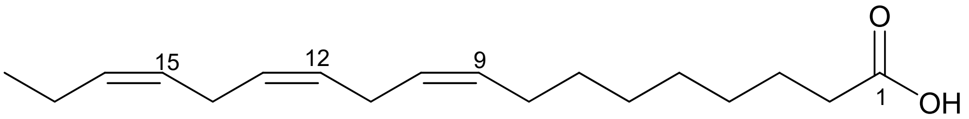

3

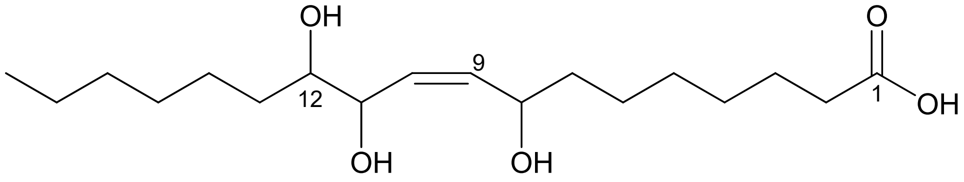

4

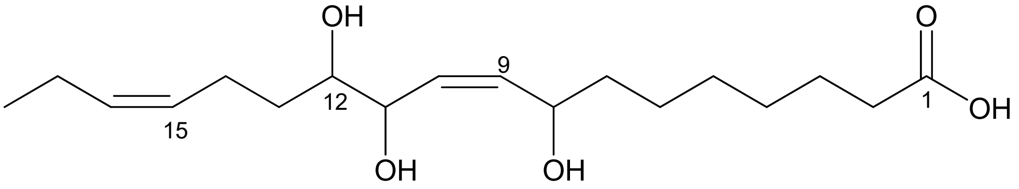

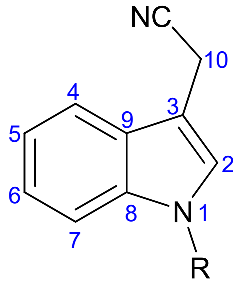

5 R=H
6 R=OCH_3

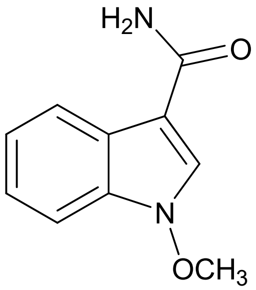

7

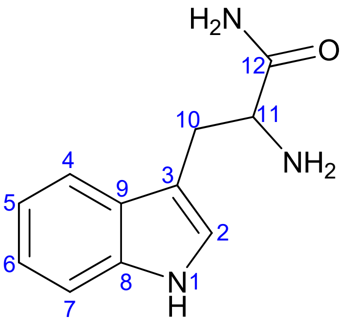

8

Figure S1. Chemical structures of the isolated compounds (**1-8**) from *Sisymbrium irio* L.

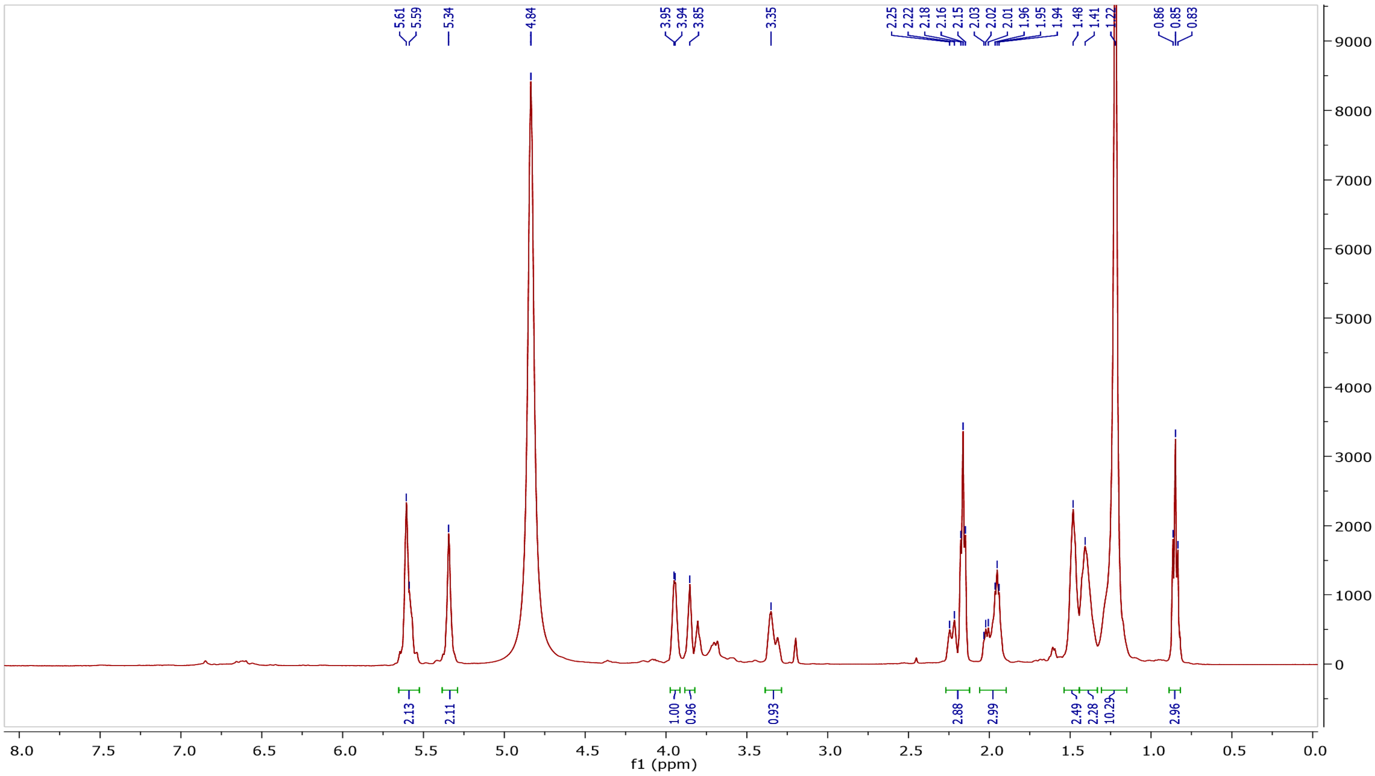

Figure S2. ^{1}H NMR spectrum of compound (**4**) (500 MHz, CD_3OD).

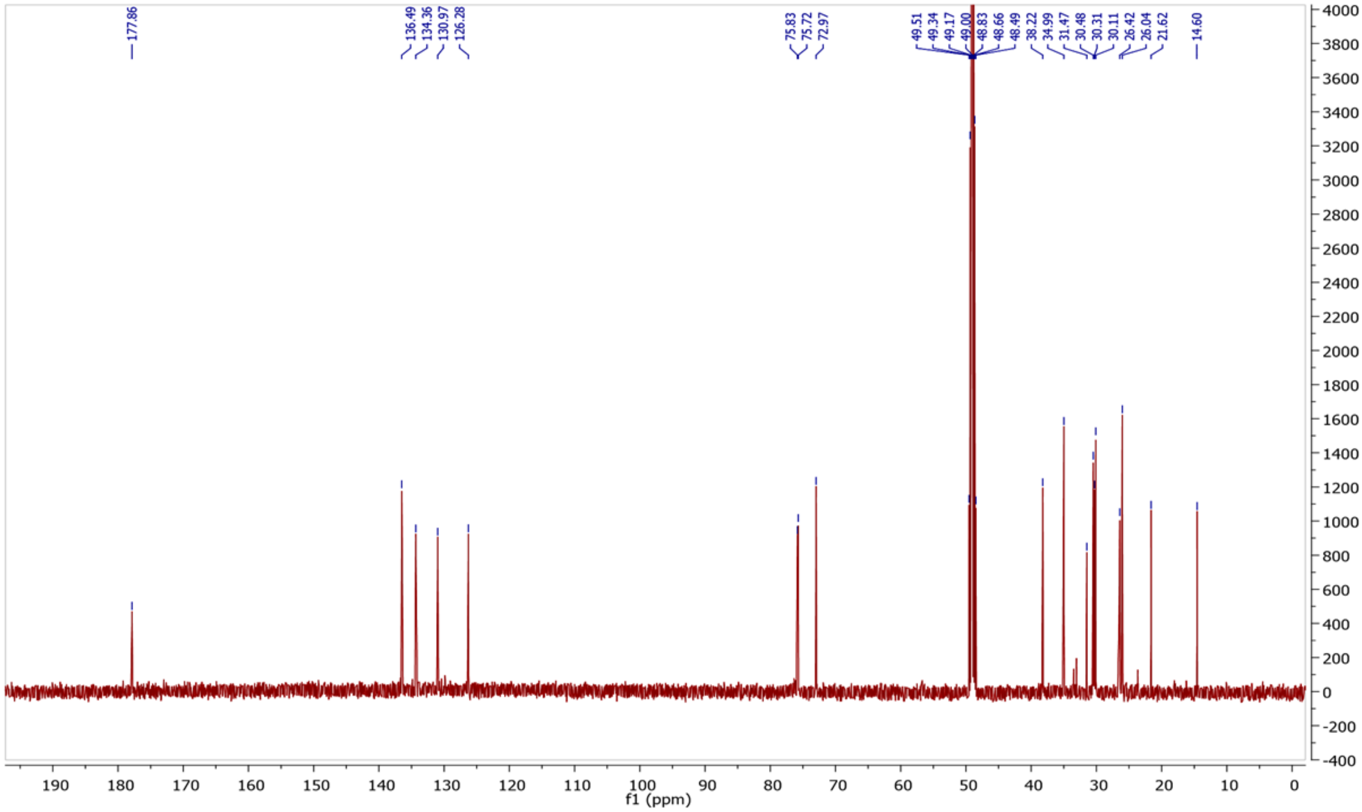

Figure S3. ^{13}C NMR spectrum of compound (**4**) (125 MHz, CD_3OD)

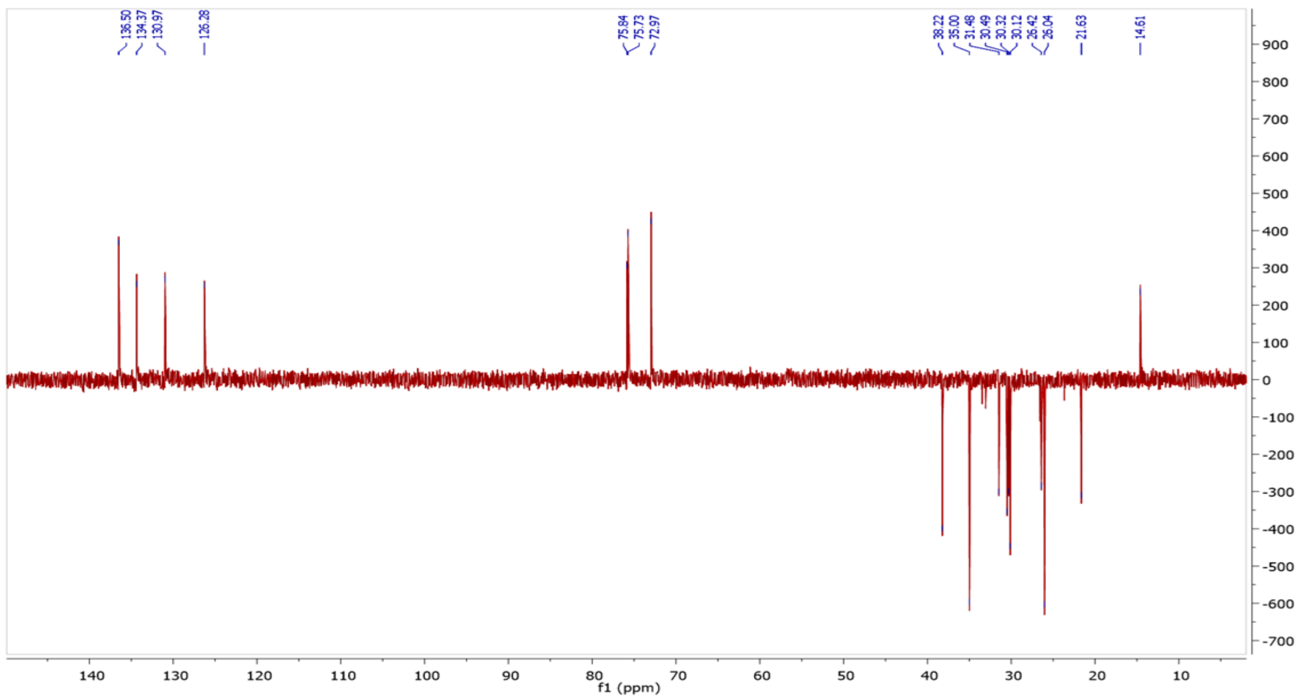

Figure S4. DEPT ^{13}C NMR spectrum of compound (**4**) (125 MHz, CD_3OD)

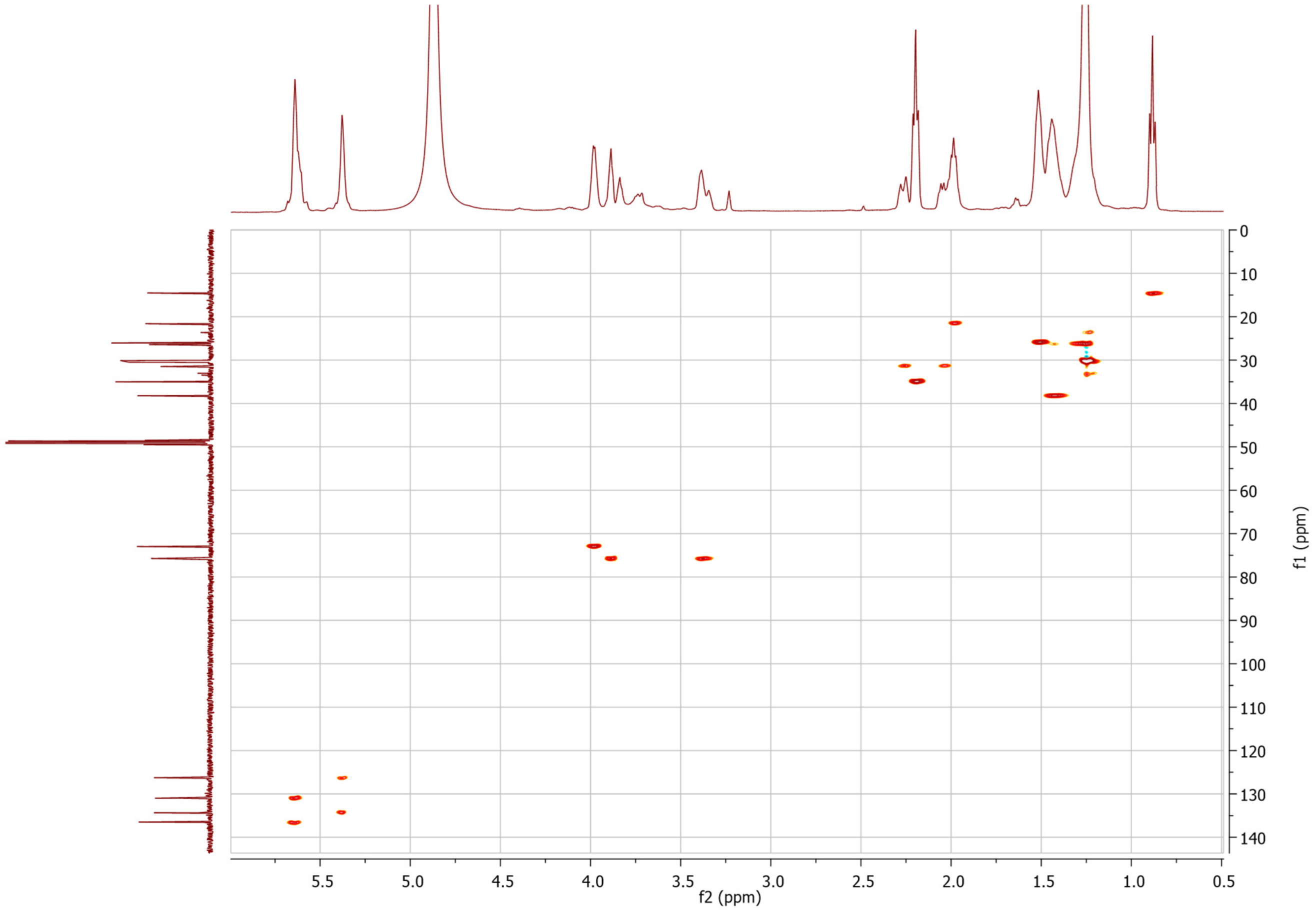

Figure S5. ^{1}H-^{13}C HSQC spectrum of compound (**4**) (500 MHz, CD_3OD)

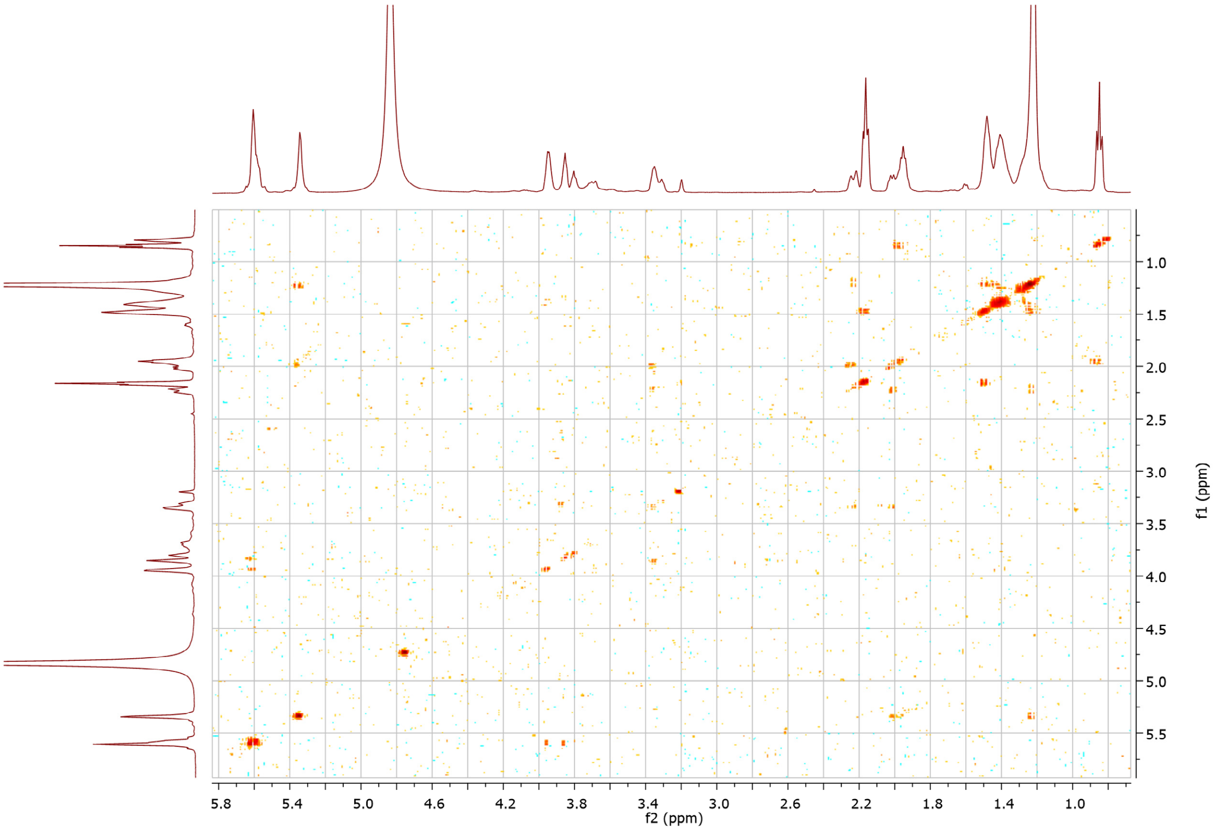

Figure S6. ^{1}H-^{1}H COSY spectrum of compound (**4**) (500 MHz, CD_3OD)

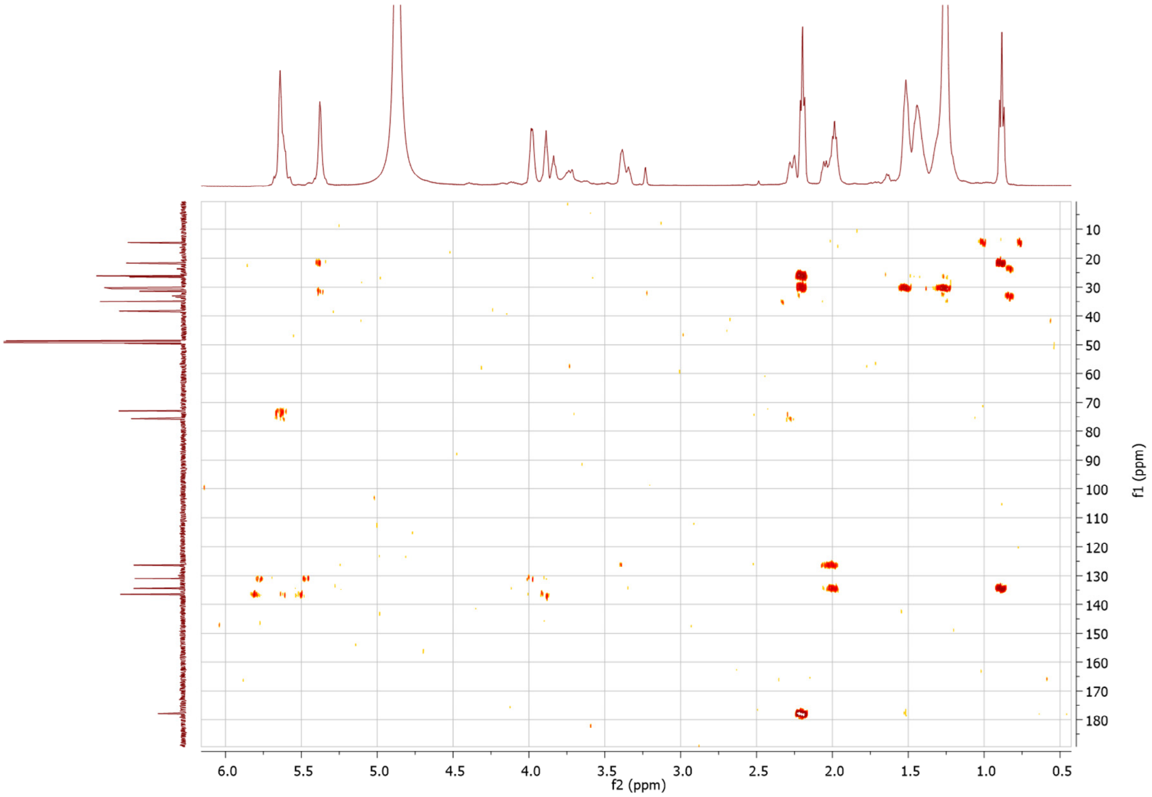

Figure S7. ^{1}H-^{13}C HMBC spectrum of compound (**4**) (500 MHz, CD_3OD)

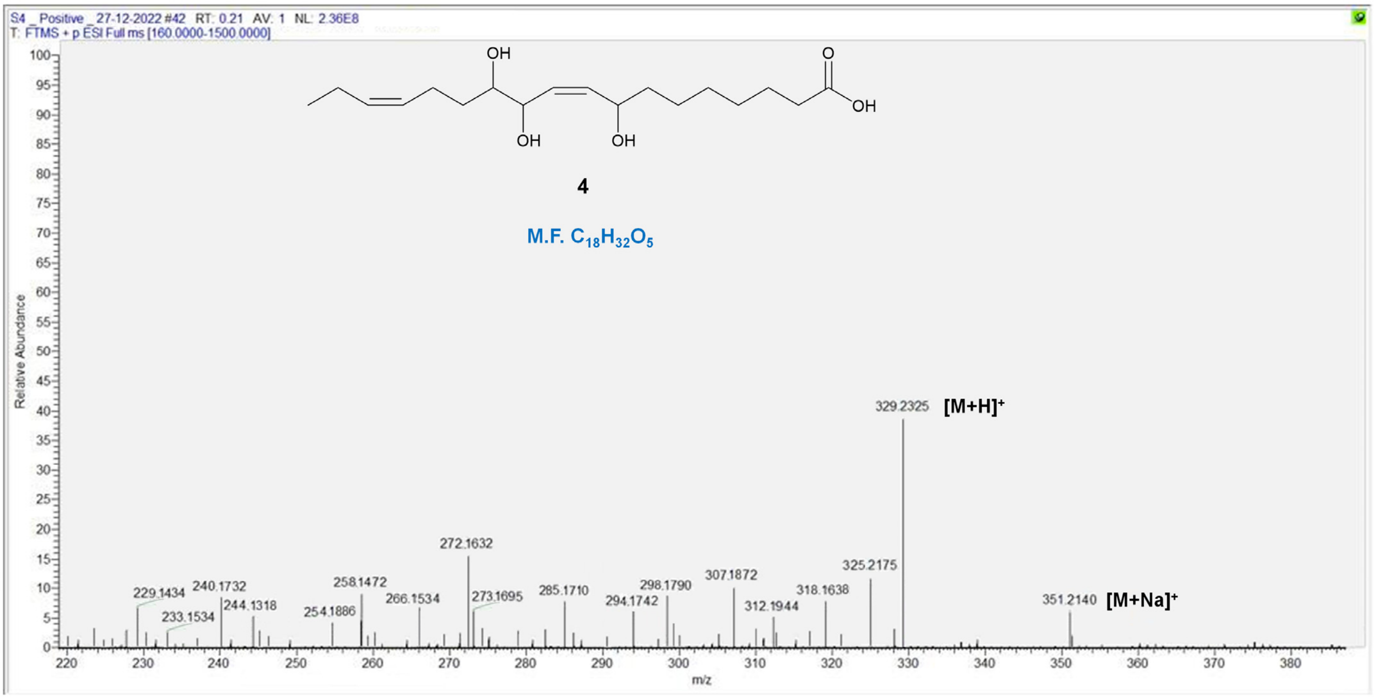

A

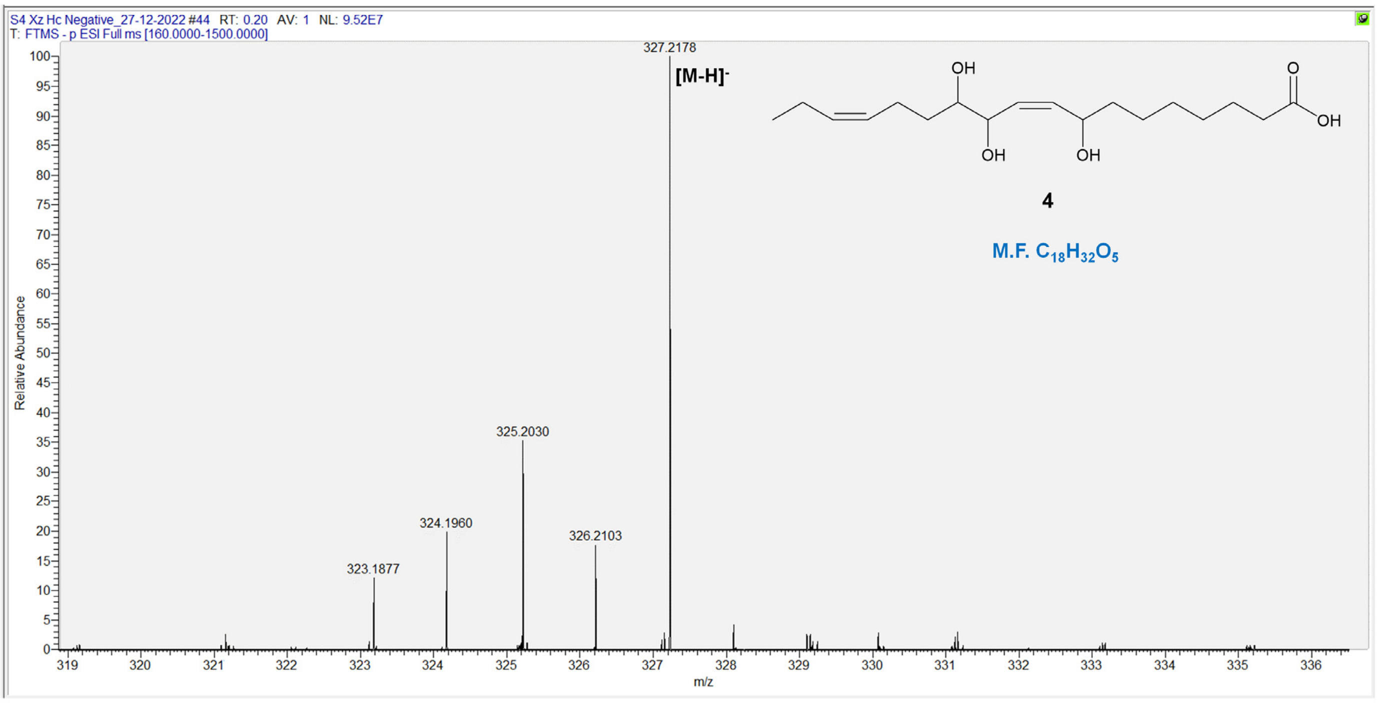

B

Figure S8. A) Positive Mode, **B**) Negative mode HRESIMS of compound (**4**).

Table S1. ^{1}H (500 MHz) and ^{13}C (125 MHz) NMR data of compounds **1**, **2** and **3**.

**No.**	1[a]		2[a]		3[b]	
	δ_H	δ_C	δ_H	δ_C	δ_H	δ_C
**1**	-	180.0	-	179.9	-	177.7
**2**	2.36, t (7.5 Hz)	34.1	2.33, *t* (7.6 Hz)	34.1	2.26, t (7.5 Hz)	34.9
**3**	1.66, *m*	24.7	1.61, *m*	24.7	1.58, brt (7.0 Hz)	26.0
**4**	1.32-1.41, *m*	28.8	1.35-1.37, *m*	29.0	1.34, m	30.1
**5**	1.32-1.41, *m*	29.4	1.35-1.37, *m*	29.1	1.34, m	30.5
**6**	2.08, *m*	27.1	1.35-1.37, *m*	29.2	1.34, m	26.4
**7**	5.39, *m*	130.0	1.35-1.37, *m*	29.6	1.51, m	38.2
**8**	5.35, *m*	127.2	2.04-2.08, *m*	27.2	4.04, dd (12.1, 6.3 Hz)	73.0
**9**	2.82, *m*	25.7	2.31-5.40, *m*	130.2	5.58, dd (9.3, 5.6 Hz)	136.5
**10**	5.37, *m*	128.3	5.31-5.40, *m*	127.8	5.58, dd (9.3, 5.6 Hz)	130.9
**11**	5.38, *m*	128.5	2.78, *m*	25.6	3.91, t (5.8 Hz)	76.5
**12**	2.82, *m*	25.7	5.31-5.40, *m*	128.21	3.41, m	75.7
**13**	5.37, *m*	128.1	5.31-5.40, *m*	128.24	1.49, m	33.0
**14**	5.40, *m*	132.1	2.78, *m*	25.5	1.34, m	26.5
**15**	2.09, *m*	20.7	5.31-5.40, *m*	127.1	1.34, m	30.3
**16**	0.99, *t* (7.6 Hz)	14.4	5.31-5.40, *m*	131.9	1.34, m	33.5
**17**	-	-	2.04-2.0,8 *m*	20.5	1.34, m	23.7
**18**	-	-	0.95 *t*, (7.6 Hz)	14.3	0.89, t (6.9 Hz)	14.5

[a] in $CDCl_3$, [b] in CD_3OD

Table S2. ^{1}H (500 MHz) and ^{13}C (125 MHz) NMR data of compounds (**5-8**).

No.	**5** [a]		**6** [a]		**7** [a]		**8** [b]	
	δ_H	δ_C	δ_H	δ_C	δ_H	δ_C	δ_H	δ_C
**1**	8.27, br s	-	-	-	-	-	11.00, s	-
**2**	7.26, br s	122.9	7.26, s	122.3	8.05, s	129.6	7.23, s	124.2
**3**	-	104.8	-	100.3	-	102.9	-	109.6
**4**	7.63, br d (5.1 Hz)	118.2	7.58, d (8.0 Hz)	118.3	8.20, d (7.8 Hz)	122.0	7.56, d (7.6 Hz)	118.4
**5**	7.23, m	120.4	7.20, t (7.8 Hz)	120.3	7.28, m	122.9	7.22, t (7.8 Hz)	118.3
**6**	7.30, m	123.0	7.33, t (7.6 Hz)	123.1	7.32, m	123.8	7.34, t (7.8 Hz)	120.9
**7**	7.44, br d (5.3 Hz)	111.7	7.48, d (8.3 Hz)	108.5	7.47, d (8.0 Hz)	123.8	7.46, d (7.8 Hz)	111.4
**8**	-	136.4	-	132.2	-	132.3	-	136.4
**9**	-	126.1	-	122.3	-	123.2	-	127.3
**10**	3.88, s	14.5	3.76, d (1.0 Hz)	13.4	-	169.8	2.97, dd (15.0, 8.9 Hz)<br>3.33, dd (15.0, 3.0 Hz)	27.1
**11**	-	118.3	-	118.0	-	-	3.47, q	54.8
**12**	-	-	-	-	-	-	-	170.4
**1-OCH3**	-	-	4.06, s	65.9	4.16, s	66.9	-	-

[a] in $CDCl_3$, [b] in DMSO-d_6

Table S3. Binding parameters for the interaction of *S. irio* compounds (**1-4**) with PPAR-γ.

Compounds	Interacting residues	Type of interaction	Bond length (Å)	Binding affinity (K_b, M^{-1})	Binding energy (ΔG, kcal mol^{-1})
**Rivoglitazone (Control)**	TYR^{473}:OH - LIG:O07	Hydrogen Bond	2.9378	1.03×10^6	-8.2
	CYS^{285}:SG - LIG	Hydrogen Bond (Pi-Donor; Pi-S)	3.6087		
	SER^{289}:OG - LIG	Hydrogen Bond (Pi-Donor)	3.6789		
	ILE^{341}:CG2 - LIG	Hydrophobic (Pi-Sigma)	3.4965		
	ILE^{341}:CG2 - LIG	Hydrophobic (Pi-Sigma)	3.8057		
	MET^{348}:SD - LIG	Pi-Sulfur	5.8190		
	MET^{348}:SD - LIG	Pi-Sulfur	5.4093		
	MET^{364}:SD - LIG	Pi-Sulfur	4.9310		
	HIS^{449} - LIG	Hydrophobic (Pi-Pi T-shaped)	4.5987		
	GLY^{284}:C,O;CYS285:N - LIG	Hydrophobic (Amide-Pi Stacked)	4.8341		
	GLY^{284}:C,O;CYS285:N - LIG	Hydrophobic (Amide-Pi Stacked)	4.3730		
	CYS^{285}:C,O;GLN286:N - LIG	Hydrophobic (Amide-Pi Stacked)	4.5366		
	LIG - CYS^{285}	Hydrophobic (Pi-Alkyl)	4.2320		
	LIG - LEU^{330}	Hydrophobic (Pi-Alkyl)	4.9590		
	LIG - CYS^{285}	Hydrophobic (Pi-Alkyl)	4.9173		
	LIG - CYS^{28}	Hydrophobic (Pi-Alkyl)	5.2276		
**Compound 1**	LIG:H - HIS^{449}:NE2	Hydrogen Bond	2.4867	8.21×10^4	-6.7
	ALA^{292} - LIG:C	Hydrophobic (Alkyl)	3.4493		
	LIG:C - MET^{329}	Hydrophobic (Alkyl)	4.0086		
	LIG:C - ILE^{296}	Hydrophobic (Alkyl)	5.1961		
	PHE^{226} - LIG:C	Hydrophobic (Alkyl)	5.1188		
**Compound 2**	LIG:H - HIS^{449}:NE2	Hydrogen Bond	2.1468	2.52×10^4	-6.0
	LIG:C - MET^{329}	Hydrophobic (Alkyl)	4.0600		
	LIG:C - LEU^{333}	Hydrophobic (Alkyl)	4.0381		
**Compound 3**	ARG^{288}:HH21 - LIG:O	Hydrogen Bond	2.4563	2.68×10^5	-7.4
	LIG:H - MET^{329}:O	Hydrogen Bond	2.1401		
	LIG:H - CYS^{285}:O	Hydrogen Bond	2.4391		
	LIG:H - CYS^{285}:O	Hydrogen Bond	2.7598		
	LIG:H – SER^{289}:OG	Hydrogen Bond	1.9331		
	LIG:C - ILE^{281}	Hydrophobic (Alkyl)	4.9626		
	LIG:C – CYS^{285}	Hydrophobic (Alkyl)	4.2712		
	PHE^{282} – LIG:C	Hydrophobic (Pi-Alkyl)	5.0299		
**Compound 4**	LIG:H - CYS^{285}:O	Hydrogen Bond	2.2347	2.98×10^4	-6.1
	LIG:H - SER^{289}:OG	Hydrogen Bond	2.2557		
	LIG:C - PHE^{282}	Hydrophobic (Pi-Sigma)	3.7546		
	ALA^{292} - LIG:C	Hydrophobic (Alkyl)	4.0911		
	LIG:C - ILE^{326}	Hydrophobic (Alkyl)	4.3909		
	LIG:C - MET^{329}	Hydrophobic (Alkyl)	4.3335		

Table S4. Binding parameters for the interaction of SI compounds (**5-8**) with 5-HT_{1A} Serotonin receptor.

**Compounds**	**Interacting residues**	**Type of interaction**	**Bond length (Å)**	**Binding affinity (K_b, M^{-1})**	**Binding energy (ΔG, kcal mol^{-1})**
**Serotonin (Control)**	LIG:H - ASP116:OD2<br>LIG:H - TYR390:OH<br>LIG:H - VAL117:O<br>LIG:H - THR121:OG1<br>LIG - PHE361<br>LIG - VAL117<br>LIG - VAL117<br>LIG - ILE189	Hydrogen Bond<br>Hydrogen Bond<br>Hydrogen Bond<br>Hydrogen Bond<br>Hydrophobic (Pi-Pi T-shaped)<br>Hydrophobic (Pi-Alkyl)<br>Hydrophobic (Pi-Alkyl)<br>Hydrophobic (Pi-Alkyl)	2.1429<br>2.2678<br>2.6567<br>1.7918<br>5.4126<br>4.1815<br>4.8280<br>4.8306	2.98×10^4	-6.1
**Compound 5**	ASP116:OD1 - LIG<br>CYS120:SG - LIG<br>CYS120:SG - LIG<br>PHE361 - LIG<br>LIG - PHE361	Electrostatic (Pi-Anion)<br>Hydrogen Bond; Pi-Sulfur<br>Hydrogen Bond<br>Hydrophobic (Pi-Pi Stacked)<br>Hydrophobic (Pi-Pi Stacked)	4.2645<br>3.6947<br>3.9737<br>3.8159<br>4.6029	4.94×10^4	-6.4
**Compound 6**	SER199:HG - LIG:N<br>VAL117:CG2 - LIG<br>PHE361 - LIG<br>LIG - VAL117<br>LIG - CYS120<br>LIG - ILE189	Hydrogen Bond<br>Hydrophobic (Pi-Sigma)<br>Hydrophobic (Pi-Pi- T-shaped)<br>Hydrophobic (Pi-Alkyl)<br>Hydrophobic (Pi-Alkyl)<br>Hydrophobic (Pi-Alkyl)	2.9146<br>3.9579<br>5.4102<br>4.6753<br>5.4746<br>5.1247	4.94×10^4	-6.4
**Compound 7**	VAL117:CG1 - LIG<br>VAL117:CG2 - LIG<br>CYS120:SG - LIG<br>LIG - ILE189<br>PHE361 - LIG	Hydrophobic (Pi-Sigma)<br>Hydrophobic (Pi-Sigma)<br>Pi-Sulfur<br>Hydrophobic (Pi-Alkyl)<br>Hydrophobic (Pi-Pi T-Shaped)	3.8512<br>3.5275<br>5.4857<br>5.1373<br>5.6271	5.85×10^4	-6.5
**Compound 8**	LIG:H - THR196:OG1<br>LIG - ILE189<br>LIG - ILE189<br>LIG - LYS191<br>LIG - ALA365<br>LIG - ALA365<br>LIG - PRO369	Hydrogen Bond<br>Hydrophobic (Pi-Alkyl)<br>Hydrophobic (Pi-Alkyl)<br>Hydrophobic (Pi-Alkyl)<br>Hydrophobic (Pi-Alkyl)<br>Hydrophobic (Pi-Alkyl)<br>Hydrophobic (Pi-Alkyl)	2.0887<br>5.2779<br>5.0783<br>5.0050<br>4.0099<br>4.9989<br>5.2075	1.15×10^5	-6.9

Table S5. Binding parameters for the interaction of *S. irio* compounds (**5-8**) with 5-HT_{2A} serotonin receptor.

Compounds	Interacting residues	Type of interaction	Bond length (Å)	Binding affinity (K_b, M^{-1})	Binding energy (ΔG, kcal mol^{-1})
**Resperidone (Control)**	SER131:HG - LIG:O	Hydrogen Bond	2.6074	4.52×10^8	-11.8
	SER159:HG - LIG:O	Hydrogen Bond	2.4294		
	ASN363:OD1 - LIG:F	Halogen (Fluorine)	3.1479		
	VAL366:CG1 - LIG	Hydrophobic (Pi-Sigma)	3.6065		
	LIG:C - TRP336	Hydrophobic (Pi-Sigma)	3.9469		
	PHE340 - LIG	Hydrophobic (Pi-Pi T-shaped)	4.7850		
	VAL366 - LIG	Hydrophobic (Alkyl)	4.5414		
	LIG - ILE163	Hydrophobic (Alkyl)	4.9503		
	PHE243 - LIG	Hydrophobic (Pi-Alkyl)	5.2906		
	PHE332 - LIG	Hydrophobic (Pi-Alkyl)	5.0258		
	TRP336 - LIG	Hydrophobic (Pi-Alkyl)	3.9891		
	PHE339 - LIG	Hydrophobic (Pi-Alkyl)	4.7884		
	PHE340 - LIG	Hydrophobic (Pi-Alkyl)	4.9599		
	LIG - VAL156	Hydrophobic (Pi-Alkyl)	5.2296		
	LIG - VAL366	Hydrophobic (Pi-Alkyl)	4.3423		
**Compound 5**	SER242:CB - LIG:O	Hydrogen Bond	3.2287	3.17×10^5	-7.5
	TRP336 - LIG	Hydrophobic (Pi-Pi T-shaped)	4.8511		
	TRP336 - LIG	Hydrophobic (Pi-Pi T-shaped)	5.2185		
	PHE340 - LIG	Hydrophobic (Pi-Pi T-shaped)	4.7955		
	LIG - PHE340	Hydrophobic (Pi-Pi T-shaped)	4.9036		
	SER159:C,O;THR160:N - LIG	Hydrophobic (Amide-Pi Stacked)	5.0640		
	SER159:C,O;THR160:N - LIG	Hydrophobic (Amide-Pi Stacked)	4.2381		
	LIG - VAL156	Pi-Alkyl	5.2206		
	LIG - ILE163	Pi-Alkyl	5.4933		
**Compound 6**	LIG:H - ASP155:OD1	Hydrogen Bond	2.7722	2.68×10^5	-7.4
	LIG:H - ASP155:OD1	Hydrogen Bond	2.9163		
	TRP336 - LIG	Hydrophobic (Pi-Pi T-shaped)	4.8407		
	TRP336 - LIG	Hydrophobic (Pi-Pi T-shaped)	5.1446		
	PHE340 - LIG	Hydrophobic (Pi-Pi T-shaped)	4.6742		
	LIG - PHE340	Hydrophobic (Pi-Pi T-shaped)	5.0964		
	SER159:C,O;THR160:N - LIG	Hydrophobic (Amide-Pi Stacked)	4.3727		
	SER159:C,O;THR160:N - LIG	Hydrophobic (Amide-Pi Stacked)	5.0037		
	LIG - VAL156	Hydrophobic (Pi-Alkyl)	5.0507		
**Compound 7**	SER159:HG - LIG:N	Hydrogen Bond	2.2083	2.60×10^5	-7.3
	LIG:H - THR160:OG1	Hydrogen Bond	2.3250		
	TRP336 - LIG	Hydrophobic (Pi-Pi T-shaped)	4.8647		
	TRP336 - LIG	Hydrophobic (Pi-Pi T-shaped)	5.3392		
	PHE340 - LIG	Hydrophobic (Pi-Pi T-shaped)	4.9159		
	LIG - PHE340	Hydrophobic (Pi-Pi T-shaped)	5.1237		
	LIG - VAL156	Hydrophobic (Pi-Alkyl)	5.1553		
	LIG - ILE163	Hydrophobic (Pi-Alkyl)	5.2300		
**Compound 8**	LIG:H - ASP155:OD1	Hydrogen Bond; Electrostatic	2.0674	2.68×10^5	-8.1
	LIG:H - THR160:OG1	Hydrogen Bond	2.5404		
	LIG:H - TYR370:OH	Hydrogen Bond	2.8946		
	LIG:H - ASP155:OD1	Hydrogen Bond	3.0856		
	LIG:H - ASP155:O	Hydrogen Bond	2.9126		
	TRP336 - LIG	Hydrophobic (Pi-Pi T-shaped)	4.8475		
	TRP336 - LIG	Hydrophobic (Pi-Pi T-shaped)	5.3792		
	PHE340 - LIG	Hydrophobic (Pi-Pi T-shaped)	4.9113		
	LIG - PHE340	Hydrophobic (Pi-Pi T-shaped)	5.0152		
	LIG - VAL156	Hydrophobic (Pi-Alkyl)	5.3190		
	LIG - ILE163	Hydrophobic (Pi-Alkyl)	5.0990		

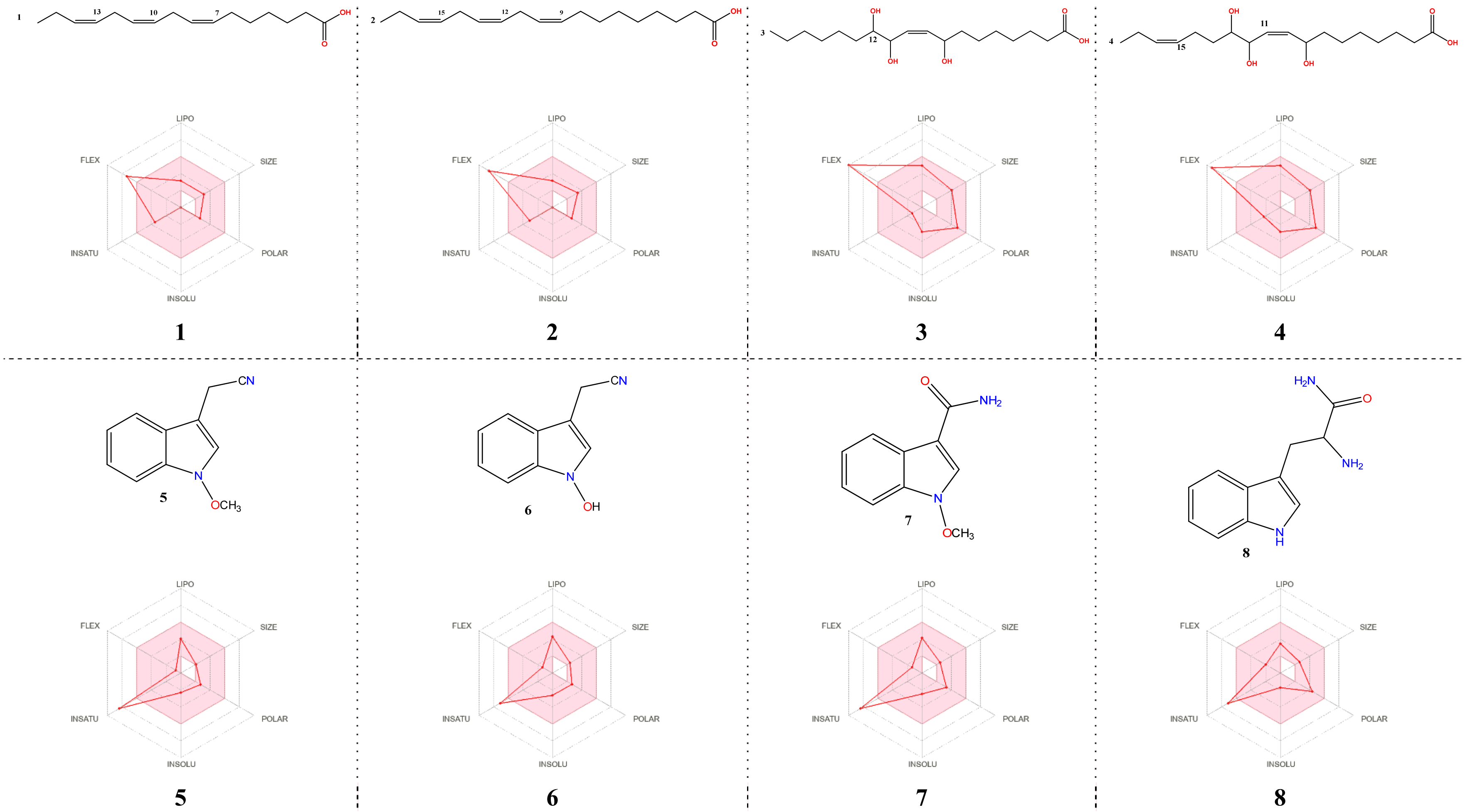

Figure S9. Bioavailability radar representations of the isolated compounds (**1-8**) from *Sisymbrium irio* L. The pink area represents the optimal range for oral bioavailability and the red line represents the optimal physicochemical properties for oral bioavailability properties.

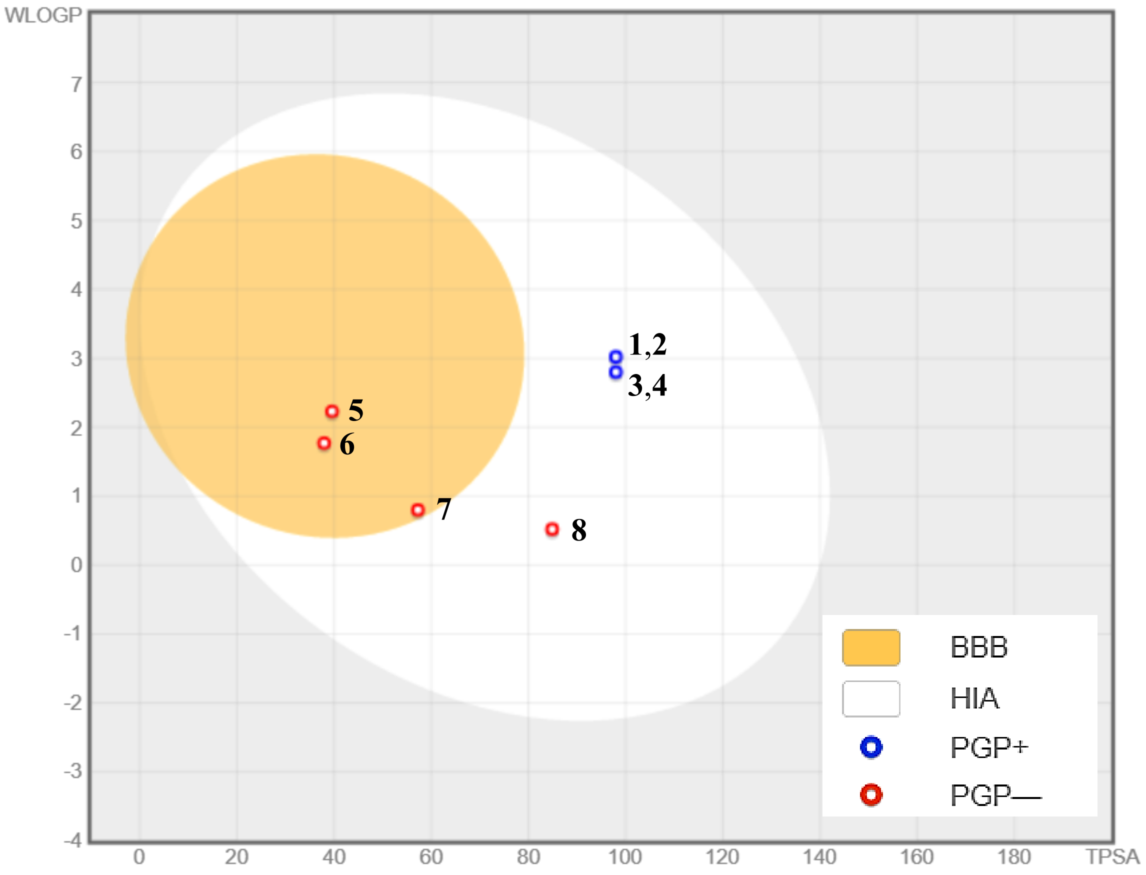

Figure S10. Boiled-egg graph of blood-brain barrier (BBB) permeability and human gastrointestinal absorption (HIA), blue circle; predicted active-efflux by P-gp, i.e., glycoprotein substrate (PGP^{+}), and red circle; no predicted active-efflux by P-gp i.e., non-substrate (PGP^{-}).

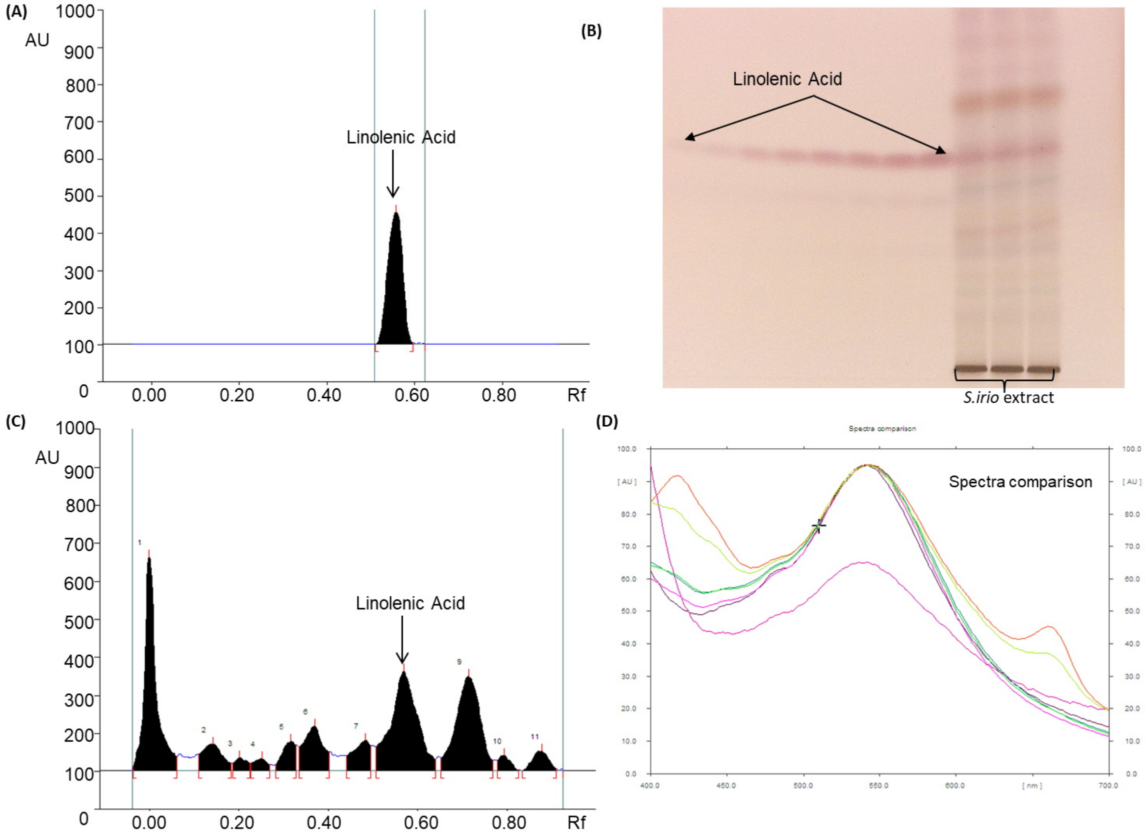

Figure S11. Chromatogram of HPTLC analysis of linolenic acid in hexane extract of *S. irio* (aerial parts) [mobile phase: acetone: n-hexane: acetic acid (25:75:0.1, v/v/v)]. (A) HPTLC chromatogram of standard linolenic acid ($R_f = 0.57 \pm 0.004$) at $\lambda_{max} = 540$ nm; (B) Pictogram of derivatized TLC plate in day light; (C) HPTLC chromatogram of *S. irio* hexane extract (linoleic acid, spot 8, $R_f = 0.57$); (D) Spectral comparison of all tracks at 540nm.
